# Supplementary figures and images for: Predicting suicide attempt or suicide death following a visit to psychiatric specialty care: A machine learning study using Swedish national registry data
Source: PLoS Med. 2020 Nov 6;17(11):e1003416. doi: 10.1371/journal.pmed.1003416 (PMC7647056; doi:10.1371/journal.pmed.1003416)

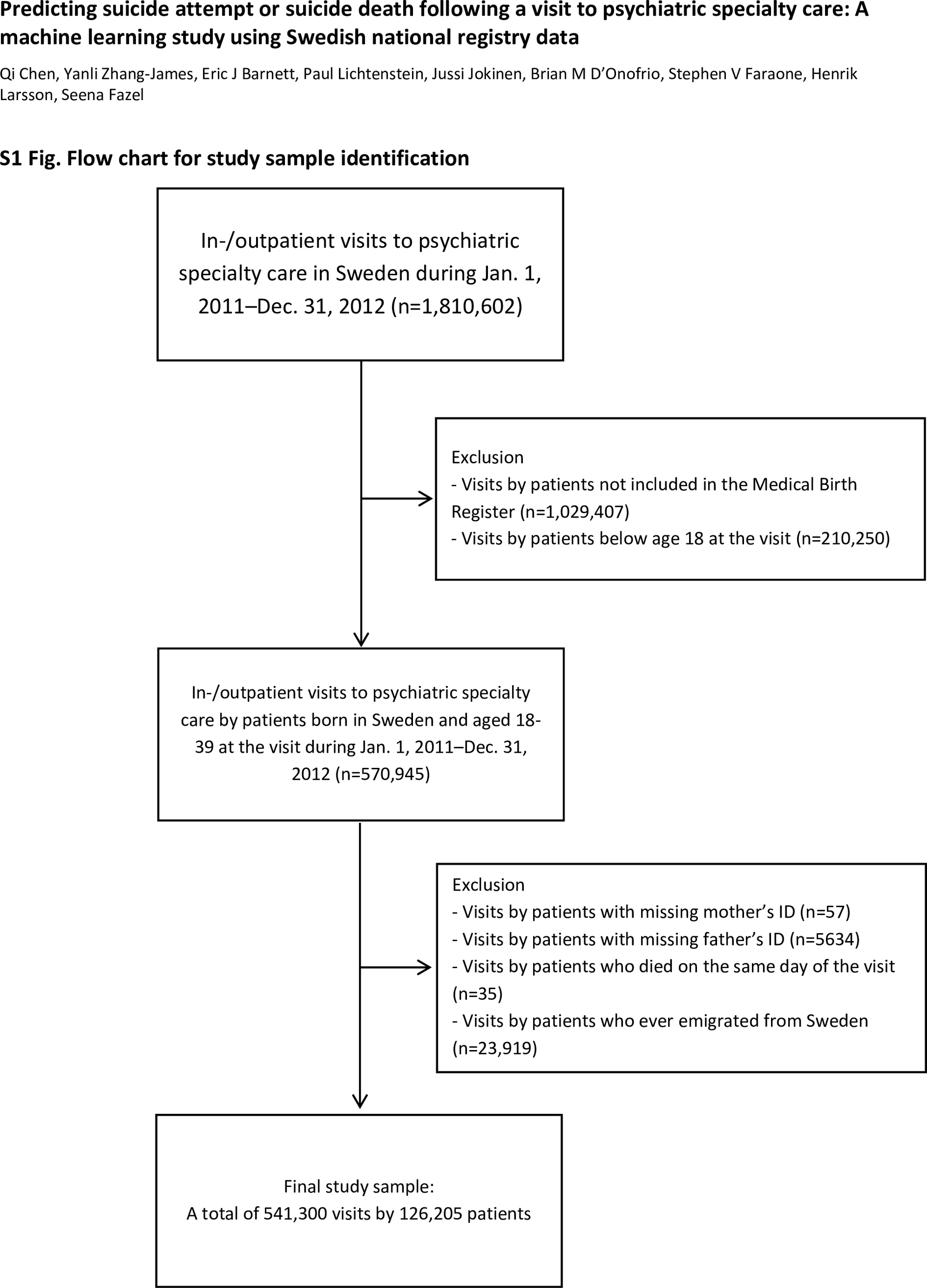

Supplement: S1 Fig — (TIF) [file pmed.1003416.s002.tif]
