# Supplementary material for: Predicting suicide attempt or suicide death following a visit to psychiatric specialty care: A machine learning study using Swedish national registry data
Source: PLoS Med. 2020 Nov 6;17(11):e1003416. doi: 10.1371/journal.pmed.1003416 (PMC7647056; doi:10.1371/journal.pmed.1003416)
Supplement: S1 Table — (DOCX) [file pmed.1003416.s003.docx]

**S1 Table. A total of 425 candidate predictors generated from the Swedish national registers**

| **The Medical Birth Register (1 predictor)** | |
| --- | --- |
| Sex (1 predictor) | |
| **The National Patient Register (299 predictors)** | |
| Age at the visit (1 predictor) | |
| Inpatient/Outpatient visit (1 predictor) | |
| Planned/unplanned visit (2 dummy indicators) | Missing (0.57%) |
| Hospitalization within 1 month prior to the visit (1 predictor) | |
| Hospitalization 1–3 months prior to the visit (1 predictor) | |
| Unplanned visit within 1 month prior to the visit (1 predictor) | |
| Unplanned visit 1–3 months prior to the visit (1 predictor) | |
| Diagnosis at the visit (37 predictors) | Intentional self-harm, Self-harm of undetermined intent, Attention-deficit/hyperactivity disorder, Substance use disorder, Intellectual disability, Autism, Anxiety disorder, Major depressive disorder, Bipolar disorders, Schizophrenia, Other psychotic disorder, Conduct disorder, Oppositional defiant disorder, Antisocial personality disorder, Borderline personality disorder, Other personality disorders, Obesity, Epilepsy, Asthma, Eczema, Hypertension, Type 1 Diabetes, Type 2 Diabetes, Parkinson disease/Parkinsonism, Ankylosing spondylitis, Celiac disease, Crohn's disease, Grave's disease, Hashimoto's disease, Multiple sclerosis, Psoriasis, Rheumatoid arthritis, Sarcoidosis, Sjogren's syndrome, Systemic lupus erythematosus, Ulcerative colitis, Accidental injuries |
| Diagnosis 1 month prior to the visit (37 predictors) |  |
| Diagnosis 1–3 months prior to the visit (37 predictors) |  |
| Diagnosis 3–6 months prior to the visit (37 predictors) |  |
| Diagnosis 6–12 months prior to the visit (37 predictors) |  |
| Diagnosis 1–3 years prior to the visit (37 predictors) |  |
| Diagnosis 3–5 years prior to the visit (37 predictors) |  |
| Method used in prior suicide attempt (32 predictors) | Poisoning, Hanging, strangulation and suffocation, Drowning and submersion, Handgun discharge, Rifle, shotgun and larger firearm discharge, Other and unspecified firearm discharge, Explosive material, Smoke, fire and flames, Steam, hot vapours and hot objects, Sharp object, Blunt object, Jumping from a high place, Jumping or lying before moving object, Crashing of motor vehicle, Other specified means, Unspecified means  Self-harm of undetermined intent by Poisoning, Hanging, strangulation and suffocation, Drowning and submersion, Handgun discharge, Rifle, shotgun and larger firearm discharge, Other and unspecified firearm discharge, Explosive material, Smoke, fire and flames, Steam, hot vapours and hot objects, Sharp object, Blunt object, Jumping from a high place, Jumping or lying before moving object, Crashing of motor vehicle, Other specified means, Unspecified means |
| **The Prescribed Drug Register (48 predictors)** | |
| Medication dispensation 1 month prior to the visit (8 predictors) | Antipsychotics, Antidepressants, Anxiolytics, Mood stabilizer, Psychostimulants, Drugs used for addictive disorders, Benzodiazepines and related drugs, Antiepileptics |
| Medication dispensation 1–3 months prior to the visit (8 predictors) |  |
| Medication dispensation 3–6 months prior to the visit (8 predictors) |  |
| Medication dispensation 6–12 months prior to the visit (8 predictors) |  |
| Medication dispensation 1–3 years prior to the visit (8 predictors) |  |
| Medication dispensation 3–5 years prior to the visit (8 predictors) |  |
| **The National Crime Register (8 predictors)** | |
| Criminal offense within 1 month prior to the visit (2 predictors) | Violent criminal offense, Non-violent criminal offense |
| Criminal offense 1–3 months prior to the visit (2 predictors) |  |
| Criminal offense 3–6 months prior to the visit (2 predictors) |  |
| Criminal offense 6–12 months prior to the visit (2 predictors) |  |
| **The Longitudinal integration database for health insurance and labor market studies (8 predictors)** | |
| Education before the visit (3 dummy indicators) | ≤9 years, 9–12 years, ≥12 years, missing (2.45%) |
| Being employed before the visit (1 predictor) | |
| Receiving study income before the visit (1 predictor) | |
| Receiving sickness or employment injury benefit before the visit (1 predictor) | |
| Receiving income support before the visit (1 predictor) | |
| Family income before the visit (1 predictor) | |
| **The Multi-generation Register + The National Patient Register (39 predictors)** | |
| Family history of diseases (39 predictors) | Intentional self-harm, Self-harm of undetermined intent, Death due to intentional self-harm, Death due to self-harm of undetermined intent, Attention-deficit/hyperactivity disorder, Substance use disorder, Intellectual disability, Autism, Anxiety disorder, Major depressive disorder, Bipolar disorders, Schizophrenia, Other psychotic disorder, Conduct disorder, Oppositional defiant disorder, Antisocial personality disorder, Borderline personality disorder, Other personality disorders, Obesity, Epilepsy, Asthma, Eczema, Hypertension, Type 1 Diabetes, Type 2 Diabetes, Parkinson disease/Parkinsonism, Ankylosing spondylitis, Celiac disease, Crohn's disease, Grave's disease, Hashimoto's disease, Multiple sclerosis, Psoriasis, Rheumatoid arthritis, Sarcoidosis, Sjogren's syndrome, Systemic lupus erythematosus, Ulcerative colitis, Accidental injuries, |
| **The Multi-generation Register + The National Crime Register (4 predictors)** | |
| Crime ever committed by parents or siblings (2 predictors) | Violent criminal offense, Non-violent criminal offense |
| Crime committed by parents or siblings within 1 year before the visit (2 predictors) |  |
| **The Multi-generation Register + The Longitudinal integration database for health insurance and labor market studies (18 predictors)** | |
| Mother’s education before the visit (3 dummy indicators) | ≤9 years, 9–12 years, ≥12 years, missing (5.27%) |
| Mother being employed before the visit (2 dummy indicators) | Missing (5.15%) |
| Mother receiving sickness or employment injury benefit before the visit (2 dummy indicators) | Missing (5.15%) |
| Mother receiving income support (2 dummy indicators) | Missing (5.15%) |
| Father’s education before the visit (3 dummy indicators) | ≤9 years, 9–12 years, ≥12 years, missing (12.48%) |
| Father being employed before the visit (2 dummy indicators) | Missing (12.24%) |
| Father receiving sickness or employment injury benefit before the visit (2 dummy indicators) | Missing (12.24%) |
| Father receiving income support (2 dummy indicators) | Missing (12.24%) |
