# Supplementary material for: Predicting suicide attempt or suicide death following a visit to psychiatric specialty care: A machine learning study using Swedish national registry data
Source: PLoS Med. 2020 Nov 6;17(11):e1003416. doi: 10.1371/journal.pmed.1003416 (PMC7647056; doi:10.1371/journal.pmed.1003416)
Supplement: S2 Table — (DOCX) [file pmed.1003416.s004.docx]

**S2 Table. International Classification of Diseases (ICD) codes for identifying clinical diagnoses from the National Patient Register**

| **Clinical diagnosis** | **ICD8** | **ICD-9 (Swedish adaption)** | **ICD-10** |
| --- | --- | --- | --- |
| Intentional self-harm | – | E950–E959 | X60–X84 |
| Self-harm of undetermined intent | – | E980–E989 | Y10–Y34 |
| Attention-deficit/hyperactivity disorder | – | 314 | F90 |
| Substance use disorder | 303, 304 | 303, 304, 305 | F10–F19 |
| Intellectual disability | 310–315 | 317, 318, 319 | F70–F73, F78, F79 |
| Autism |  | 299 | F84 |
| Anxiety disorder | 300 (except 300.4) | 300 (except 300E) | F40, F41, F42, F44, F45, F48 |
| Major depressive disorder | 296.2 | 296B | F32, F33 |
| Bipolar disorders | 296.1, 296.3, 296.8 | 296A, 296C, 296D, 296E, 296W | F30, F31 |
| Schizophrenia | 295 | 295 | F20 |
| Other psychotic disorder | 297, 298.2, 298.3, 298.4, 298.8, 298.9, 299 | 297, 298 | F21–F29 |
| Conduct disorder | – | 312 | F91 |
| Oppositional defiant disorder |  | 313W | F913 |
| Antisocial personality disorder | 301.7 | 301H | F60.2 |
| Borderline personality disorder | 301.3, 301.5, 306.9 | 301D, 301J | F60.3 |
| Other personality disorders | 300.9, 301.0–301.2, 301.4, 301.6–301.9, 307 | 301A–301C, 301E–301H, 301J, 301W, 301X | F60.0, F60.1, F60.4–F60.9, F61.9 |
| Obesity | 277.99 | 278A, 278B | E65, E66 |
| Epilepsy | 345 | 345 | G40, G41 |
| Asthma | 493 | 493 | J45, J46 |
| Eczema | 690–692 | 690–693 | L20–L27, L30 |
| Hypertension | 400-404 | 401-405 | I10-I15 |
| Type 1 Diabetes | – | – | E10 |
| Type 2 Diabetes | – | – | E11 |
| Parkinson disease/Parkinsonism | 342 | 332E | G20, G21, G22 |
| Ankylosing spondylitis | 712.4 | 720A | M45 |
| Celiac disease | 269.00, 269.99 | 579A | K90.0 |
| Crohn's disease | 563 | 555 | K50 |
| Grave's disease | 242 | 242A | E05.0 |
| Hashimoto's disease | 245.03 | 245C | E06.3 |
| Multiple sclerosis | 340 | 340 | G35 |
| Psoriasis | 696.10, 696.19 | 696B | L40.0–L40.3, L40.8, L40.9 |
| Rheumatoid arthritis | 712.10, 712.38, 712.39, 712.50, 712.59 | 714A, 714B, 714C | M05, M06 |
| Sarcoidosis | 135 | 135 | D86 |
| Sjogren's syndrome | – | 710C | M35.0 |
| Systemic lupus erythematosus | 734.1 | 710A | M32 |
| Ulcerative colitis | 563.1 | 556 | K51 |
| Accidental injuries | (800–929, 950–959, 996) &  (E807–E846, E880–E887, E916–E918) | (800–904, 910–929, 950–957, 959) &  (E807–E849, E880–E888, E916–E920, E922) | (S00–T14) & (V01–W34) |
