# Supplementary material for: Predicting suicide attempt or suicide death following a visit to psychiatric specialty care: A machine learning study using Swedish national registry data
Source: PLoS Med. 2020 Nov 6;17(11):e1003416. doi: 10.1371/journal.pmed.1003416 (PMC7647056; doi:10.1371/journal.pmed.1003416)
Supplement: S3 Table — (DOCX) [file pmed.1003416.s005.docx]

**S3 Table. International Classification of Diseases (ICD)-10 codes for identifying methods used for prior self-harm from the National Patient Register**

| **Intentional self-harm** | **ICD-10** | **Self-harm of undetermined intent** | **ICD-10** |
| --- | --- | --- | --- |
| Poisoning | X6* | Poisoning | Y1* |
| Hanging, strangulation and suffocation | X70 | Hanging, strangulation and suffocation | Y20 |
| Drowning and submersion | X71 | Drowning and submersion | Y21 |
| Handgun discharge | X72 | Handgun discharge | Y22 |
| Rifle, shotgun and larger firearm discharge | X73 | Rifle, shotgun and larger firearm discharge | Y23 |
| Other and unspecified firearm discharge | X74 | Other and unspecified firearm discharge | Y24 |
| Explosive material | X75 | Explosive material | Y25 |
| Smoke, fire and flames | X76 | Smoke, fire and flames | Y26 |
| Steam, hot vapours and hot objects | X77 | Steam, hot vapours and hot objects | Y27 |
| Sharp object | X78 | Sharp object | Y28 |
| Blunt object | X79 | Blunt object | Y29 |
| Jumping from a high place | X80 | Jumping from a high place | Y30 |
| Jumping or lying before moving object | X81 | Jumping or lying before moving object | Y31 |
| Crashing of motor vehicle | X82 | Crashing of motor vehicle | Y32 |
| Other specified means | X83 | Other specified means | Y33 |
| Unspecified means | X84 | Unspecified means | Y34 |
