# Supplementary material for: Predicting suicide attempt or suicide death following a visit to psychiatric specialty care: A machine learning study using Swedish national registry data
Source: PLoS Med. 2020 Nov 6;17(11):e1003416. doi: 10.1371/journal.pmed.1003416 (PMC7647056; doi:10.1371/journal.pmed.1003416)
Supplement: S4 Table — (DOCX) [file pmed.1003416.s006.docx]

**S4 Table. Prior use of medications identified from the Prescribed Drug Register according to the Anatomical Therapeutic Chemical (ATC) Classification System**

| **Medication** | **ATC codes** |
| --- | --- |
| Antipsychotics | N05A* |
| Antidepressants | N06A* |
| Anxiolytics | N05BB*, N05BE*, N05CH*, N05CM* |
| Mood stabilizer | N05AN* |
| Psychostimulants | N06B* |
| Drugs used for addictive disorders | N07BB01, N07BB03, N07BB04, N02AE01, N07BC01, N07BC02, N07BC51 |
| Benzodiazepines and related drugs | N03AE*, N05BA*, N05CD*, N05CF* |
| Antiepileptics | N03A* |

***** A total of 48 predictors were generated for each selected category of medications within different time windows (i.e., 1 month, 1–3 months, 3–6 months, 6–12 months, 1–3 years, 3–5 years prior to the visit).
