# Supplementary material for: Predicting suicide attempt or suicide death following a visit to psychiatric specialty care: A machine learning study using Swedish national registry data
Source: PLoS Med. 2020 Nov 6;17(11):e1003416. doi: 10.1371/journal.pmed.1003416 (PMC7647056; doi:10.1371/journal.pmed.1003416)
Supplement: S5 Table — (DOCX) [file pmed.1003416.s007.docx]

**S5 Table. Training and validation AUCs obtained from 10-fold cross validation for the best models trained by each selected machine learning algorithm and the ensemble of different combinations of these best models**

|  | **Suicide attempt within 90 days** | | | | |  | **Suicide attempt within 30 days** | | | | |
| --- | --- | --- | --- | --- | --- | --- | --- | --- | --- | --- | --- |
|  | **Training AUC** | |  | **Validation AUC** | |  | **Training AUC** | |  | **Validation AUC** | |
| **Model** | **Mean** | **SD** |  | **Mean** | **SD** |  | **Mean** | **SD** |  | **Mean** | **SD** |
| EN | 0.8763 | 0.0016 |  | 0.8721 | 0.0163 |  | 0.8918 | 0.0018 |  | 0.8883 | 0.0147 |
| RF | 0.8783 | 0.0017 |  | 0.8703 | 0.0154 |  | 0.8984 | 0.0013 |  | 0.8875 | 0.0145 |
| GB | 0.8820 | 0.0017 |  | 0.8741 | 0.0164 |  | 0.8987 | 0.0016 |  | 0.8902 | 0.0152 |
| NN | 0.8789 | 0.0016 |  | 0.8735 | 0.0158 |  | 0.8925 | 0.0016 |  | 0.8878 | 0.0144 |
| EN+RF | 0.8798 | 0.0016 |  | 0.8737 | 0.0156 |  | 0.8975 | 0.0015 |  | 0.8898 | 0.0144 |
| EN+GB | 0.8810 | 0.0016 |  | 0.8747 | 0.0161 |  | 0.8969 | 0.0016 |  | 0.8906 | 0.0147 |
| EN+NN | 0.8781 | 0.0016 |  | 0.8733 | 0.0159 |  | 0.8929 | 0.0016 |  | 0.8888 | 0.0145 |
| RF+GB | 0.8823 | 0.0016 |  | 0.8742 | 0.0157 |  | 0.9008 | 0.0014 |  | 0.8907 | 0.0145 |
| RF+NN | 0.8808 | 0.0016 |  | 0.8742 | 0.0154 |  | 0.8979 | 0.0014 |  | 0.8898 | 0.0141 |
| GB+NN | 0.8819 | 0.0016 |  | 0.8751 | 0.0160 |  | 0.8972 | 0.0016 |  | 0.8904 | 0.0146 |
| EN+RF+GB | 0.8819 | 0.0016 |  | 0.8749 | 0.0157 |  | 0.8993 | 0.0015 |  | 0.8910 | 0.0144 |
| EN+GB+NN | 0.8808 | 0.0016 |  | 0.8747 | 0.0159 |  | 0.8962 | 0.0016 |  | 0.8904 | 0.0145 |
| EN+RF+NN | 0.8803 | 0.0016 |  | 0.8745 | 0.0156 |  | 0.8970 | 0.0015 |  | 0.8901 | 0.0142 |
| RF+GB+NN | 0.8824 | 0.0016 |  | 0.8751 | 0.0156 |  | 0.8995 | 0.0015 |  | 0.8910 | 0.0142 |
| EN+RF+GB+NN | 0.8817 | 0.0016 |  | 0.8751 | 0.0156 |  | 0.8985 | 0.0015 |  | 0.8910 | 0.0143 |

AUC: area under the receiver operating characteristic curve

EN: The best elastic net penalized logistic regression model

RF: The best random forest model

GB: The best gradient boosting model

NN: The best neural network model
