# Supplementary material for: Predicting suicide attempt or suicide death following a visit to psychiatric specialty care: A machine learning study using Swedish national registry data
Source: PLoS Med. 2020 Nov 6;17(11):e1003416. doi: 10.1371/journal.pmed.1003416 (PMC7647056; doi:10.1371/journal.pmed.1003416)
Supplement: S6 Table — (DOCX) [file pmed.1003416.s008.docx]

**S6 Table. Optimization of hyperparameters using grid search**

| **Model** | **Hyperparameter** | **Range of hyperparameter in grid search** | **Selected value** |
| --- | --- | --- | --- |
| Elastic net | alpha | 0.0001, 0.0005, 0.001, 0.005, 0.01, 0.03, 0.05 | 0.001 |
|  | l1_ratio | 0.01, 0.03, 0.05, 0.07, 0.09 | 0.05 |
|  | penalty | 'elasticnet' | 'elasticnet' |
| Random forest | criterion | 'entropy' | 'entropy' |
|  | max_features | 0.01, 0.02, 0.03, 0.05, 0.07, 0.09, 0.1 | 0.05 |
|  | n_estimators | 100, 200, 300, 400, 500 | 100 |
|  | max_depth | 3, 5, 7, 9, 11 | 7 |
| Gradient boosting | n_estimators | 50, 100, 200, 300 | 100 |
|  | learning_rate | 0.05, 0.1, 0.3 | 0.1 |
|  | subsample | 0.5, 0.7, 0.9 | 0.7 |
|  | max_depth | 3, 4, 5, 6, 7 | 3 |
|  | colsample_bytree | 0.5, 0.6, 0.7, 0.8, 0.9 | 0.5 |
|  | gamma | 1 | 1 |
| Neural network | solver | 'sgd' | 'sgd' |
|  | hidden_layer_sizes | (6, 1), (6, 2), (7, 1), (7, 2), (8, 1), (8, 2) | (7,2) |
|  | alpha | 0.5, 0.6, 0.7 | 0.6 |

*Default values were used for hyperparameters that are not listed.
