# Supplementary material for: Predicting suicide attempt or suicide death following a visit to psychiatric specialty care: A machine learning study using Swedish national registry data
Source: PLoS Med. 2020 Nov 6;17(11):e1003416. doi: 10.1371/journal.pmed.1003416 (PMC7647056; doi:10.1371/journal.pmed.1003416)
Supplement: S7 Table — predicted outcome at the 95th percentile risk threshold. (DOCX) [file pmed.1003416.s009.docx]

**S7 Table. Number of true vs. predicted outcome at the 95^th^ percentile risk threshold**

|  | **Outcome_90_ (+)** | **Outcome_90_(–)** | **Total** | **Outcome_30_ (+)** | **Outcome_30_ (–)** | **Total** |
| --- | --- | --- | --- | --- | --- | --- |
| **Predicted (+)** | 1892 | 3522 | 5414 | 1010 | 4404 | 5414 |
| **Predicted (–)** | 2115 | 100,747 | 102,862 | 901 | 101,961 | 102,862 |
| **Total** | 4007 | 104,269 | 108,276 | 1911 | 106,365 | 108,276 |

Outcome_90_: suicide attempt/death within 90 days following a visit to psychiatric specialty care

Outcome_30_: suicide attempt/death within 30 days following a visit to psychiatric specialty care

The results were based on ensemble models
