# Supplementary material for: Predicting suicide attempt or suicide death following a visit to psychiatric specialty care: A machine learning study using Swedish national registry data
Source: PLoS Med. 2020 Nov 6;17(11):e1003416. doi: 10.1371/journal.pmed.1003416 (PMC7647056; doi:10.1371/journal.pmed.1003416)
Supplement: S8 Table — (DOCX) [file pmed.1003416.s010.docx]

**S8 Table. Model performance metrics at various risk thresholds for predicting suicide attempt/death within 90 and 30 days following a visit to psychiatric specialty care during 2011–2012, predictors being restricted to sex, age at the visit, and those identified from the National Patient Register as well as the Prescribed Drug Register.**

| **Risk threshold** | **Sensitivity (%)** | **Specificity (%)** | **PPV (%)** | **NPV (%)** |
| --- | --- | --- | --- | --- |
| *Suicide attempt/death within 90 days following a visit* | | | | |
| 99.5^th^ | 9.8 | 99.9 | 72.0 | 96.6 |
| 99^th^ | 17.3 | 99.6 | 64.1 | 96.9 |
| 98^th^ | 27.8 | 99.0 | 51.3 | 97.3 |
| 97^th^ | 36.1 | 98.3 | 44.5 | 97.6 |
| 96^th^ | 41.7 | 97.4 | 38.6 | 97.8 |
| 95^th^ | 46.6 | 96.6 | 34.5 | 97.9 |
| 90^th^ | 60.9 | 92.0 | 22.6 | 98.4 |
| 85^th^ | 69.6 | 87.1 | 17.2 | 98.7 |
| 80^th^ | 75.6 | 82.1 | 14.0 | 98.9 |
| 70^th^ | 82.7 | 72.0 | 10.2 | 99.1 |
| 60^th^ | 87.7 | 61.8 | 8.1 | 99.2 |
| 50^th^ | 91.5 | 51.6 | 6.8 | 99.4 |
| *Suicide attempt/death within 30 days following a visit* | | | | |
| 99.5^th^ | 12.7 | 99.7 | 44.6 | 98.5 |
| 99^th^ | 21.3 | 99.4 | 37.6 | 98.6 |
| 98^th^ | 34.4 | 98.6 | 30.4 | 98.8 |
| 97^th^ | 41.9 | 97.7 | 24.7 | 98.9 |
| 96^th^ | 47.8 | 96.8 | 21.1 | 99.0 |
| 95^th^ | 51.5 | 95.8 | 18.2 | 99.1 |
| 90^th^ | 65.9 | 91.0 | 11.6 | 99.3 |
| 85^th^ | 73.7 | 86.1 | 8.7 | 99.5 |
| 80^th^ | 78.8 | 81.1 | 7.0 | 99.5 |
| 70^th^ | 85.3 | 71.0 | 5.0 | 99.6 |
| 60^th^ | 89.7 | 60.9 | 4.0 | 99.7 |
| 50^th^ | 93.0 | 50.8 | 3.3 | 99.8 |
| PPV: Positive predictive value; NPV: Negative predictive value | | | |  |
