# Supplementary material for: Predicting suicide attempt or suicide death following a visit to psychiatric specialty care: A machine learning study using Swedish national registry data
Source: PLoS Med. 2020 Nov 6;17(11):e1003416. doi: 10.1371/journal.pmed.1003416 (PMC7647056; doi:10.1371/journal.pmed.1003416)
Supplement: S9 Table — (DOCX) [file pmed.1003416.s011.docx]

**S9 Table. Statistics underlying model calibration curves for predicting suicide attempt/death within 90 and 30 days following a visit to psychiatric specialty care during 2011–2012**

| **Bin** | **Range of predicted risk** | **Number of unique individuals** | **Number of index visits** | **Number of positives** | **Mean predicted risk** | **Observed proportion of true positives** |
| --- | --- | --- | --- | --- | --- | --- |
| *Suicide attempt/death within 90 days following a visit* | | | | | |  |
| 1 | [0.000, 0.111) | 25,130 | 100,816 | 1858 | 0.0171 | 0.0184 |
| 2 | [0.111, 0.222) | 962 | 3966 | 643 | 0.1535 | 0.1621 |
| 3 | [0.222, 0.333) | 340 | 1081 | 309 | 0.2684 | 0.2858 |
| 4 | [0.333, 0.444) | 248 | 992 | 356 | 0.3918 | 0.3589 |
| 5 | [0.444, 0.556) | 136 | 599 | 295 | 0.4950 | 0.4925 |
| 6 | [0.556, 0.667) | 78 | 383 | 217 | 0.5969 | 0.5666 |
| 7 | [0.667, 0.778) | 52 | 327 | 232 | 0.7124 | 0.7095 |
| 8 | [0.778, 0.889) | 21 | 81 | 66 | 0.8026 | 0.8148 |
| 9 | [0.889, 1.000] | 4 | 31 | 31 | 0.9415 | 1.0000 |
| *Suicide attempt/death within 30 days following a visit* | | | | | |  |
| 1 | [0.000, 0.125) | 25,223 | 105,266 | 1130 | 0.0101 | 0.0107 |
| 2 | [0.125, 0.250) | 354 | 1614 | 300 | 0.1909 | 0.1859 |
| 3 | [0.250, 0.375) | 146 | 837 | 239 | 0.3102 | 0.2855 |
| 4 | [0.375, 0.500) | 66 | 423 | 172 | 0.4301 | 0.4066 |
| 5 | [0.500, 0.625) | 25 | 96 | 48 | 0.5528 | 0.5000 |
| 6 | [0.625, 0.750) | 4 | 40 | 22 | 0.6614 | 0.5500 |
| 7 | [0.750, 0.875) | 0 | 0 | 0 | NA | NA |
| 8 | [0.875, 1.000] | 0 | 0 | 0 | NA | NA |

Test set was divided into a number of bins based on the predicted risk. No value was returned for bins without index visits.
