# Supplementary material for: Predicting suicide attempt or suicide death following a visit to psychiatric specialty care: A machine learning study using Swedish national registry data
Source: PLoS Med. 2020 Nov 6;17(11):e1003416. doi: 10.1371/journal.pmed.1003416 (PMC7647056; doi:10.1371/journal.pmed.1003416)
Supplement: S10 Table — (DOCX) [file pmed.1003416.s012.docx]

**S10 Table. Elastic net logistic regression model selected predictors and coefficients.**

| **Suicide attempt within 90 days** | | **Suicide attempt within 30 days** | |
| --- | --- | --- | --- |
| **Predictor/Intercept** | **coefficient** | **Predictor/Intercept** | **coefficient** |
| Intercept | -5.44765338 | Intercept | -5.62078962 |
| Intentional self-harm 3–6 months prior to the visit | 0.58615284 | Intentional self-harm 3–6 months prior to the visit | 0.51326524 |
| Unplanned visit | 0.55383830 | Intentional self-harm 1 month prior to the visit | 0.50914670 |
| Intentional self-harm 1 month prior to the visit | 0.54899243 | Intentional self-harm 1–3 months prior to the visit | 0.50838532 |
| Intentional self-harm 1–3 months prior to the visit | 0.54381869 | Intentional self-harm by Sharp object | 0.42191518 |
| Intentional self-harm 6–12 months prior to the visit | 0.42724267 | Intentional self-harm by Poisoning | 0.41936046 |
| Schizophrenia in relatives | 0.42662724 | Schizophrenia in relatives | 0.40886858 |
| Intentional self-harm by Sharp object | 0.38910350 | Unplanned visit | 0.38838903 |
| Intentional self-harm by Poisoning | 0.38840546 | Intentional self-harm 6–12 months prior to the visit | 0.37384959 |
| Unplanned visit 1–3 months prior to the visit | 0.35658930 | Unplanned visit within 1 month prior to the visit | 0.30474443 |
| Unplanned visit within 1 month prior to the visit | 0.33421342 | Hospitalization within 1 month prior to the visit | 0.29323055 |
| Age at index visit | -0.32629008 | Unplanned visit 1–3 months prior to the visit | 0.28411652 |
| Hospitalization within 1 month prior to the visit | 0.24203187 | Planned visit | -0.28295888 |
| Substance use disorder in relatives | 0.23107663 | Age at index visit | -0.24443076 |
| Intentional self-harm 1–3 years prior to the visit | 0.21150726 | Intentional self-harm in relatives | 0.21839934 |
| Intentional self-harm in relatives | 0.20853749 | Borderline personality disorder in relatives | 0.21570725 |
| Borderline personality disorder in relatives | 0.20597466 | Intentional self-harm 1–3 years prior to the visit | 0.18392619 |
| Major depressive disorder 1 month prior to the visit | 0.20345441 | Intentional self-harm at the visit | 0.17725880 |
| Intentional self-harm at the visit | 0.17762161 | Substance use disorder in relatives | 0.17520179 |
| Hospitalization 1–3 months prior to the visit | 0.17592583 | Hospitalization 1–3 months prior to the visit | 0.17335219 |
| Anxiety disorder 1 month prior to the visit | 0.16760119 | Anxiety disorder 1 month prior to the visit | 0.14884991 |
| Receiving study income before the visit | 0.16282027 | Anxiolytics dispensation 6–12 months prior to the visit | 0.14501811 |
| Sex | 0.16122164 | Anxiety disorder in relatives | 0.14006635 |
| Attention-deficit/hyperactivity disorder at the visit | -0.15350370 | Sex | 0.13839069 |
| Major depressive disorder in relatives | 0.15337339 | Major depressive disorder 1 month prior to the visit | 0.13031792 |
| Benzodiazepines and related drugs dispensation 6–12 months prior to the visit | 0.14080895 | Major depressive disorder in relatives | 0.12586707 |
| Substance use disorder 1 month prior to the visit | -0.12851450 | Inpatient visit | -0.12543858 |
| Anxiety disorder in relatives | 0.12808340 | Attention-deficit/hyperactivity disorder at the visit | -0.12075092 |
| Benzodiazepines and related drugs dispensation 1 month prior to the visit | 0.12584067 | Antipsychotics dispensation 1–3 years prior to the visit | -0.11889676 |
| Other personality disorders 6–12 months prior to the visit | 0.12583416 | Receiving study income before the visit | 0.11809570 |
| Education before the visit ≤9 years | 0.12271210 | Benzodiazepines and related drugs dispensation 6–12 months prior to the visit | 0.11081268 |
| Anxiolytics dispensation 6–12 months prior to the visit | 0.12210504 | Other personality disorders 6–12 months prior to the visit | 0.10656782 |
| Anxiety disorder 3–5 years prior to the visit | -0.12069950 | Benzodiazepines and related drugs dispensation 1 month prior to the visit | 0.10450272 |
| Borderline personality disorder at the visit | 0.11934232 | Substance use disorder 3–6 months prior to the visit | 0.09552884 |
| Intentional self-harm by Unspecified means | 0.11392485 | Accidental injuries 6–12 months prior to the visit | 0.09550222 |
| Major depressive disorder 3–6 months prior to the visit | 0.11056072 | Intentional self-harm by Unspecified means | 0.09120022 |
| Inpatient visit | -0.10986328 | Father’s education before the visit 9–12 years | 0.09079606 |
| Autism 1 month prior to the visit | 0.10798089 | Accidental injuries 1 month prior to the visit | 0.09074999 |
| Accidental injuries 1 month prior to the visit | 0.10581916 | Major depressive disorder 3–6 months prior to the visit | 0.09038932 |
| Substance use disorder 3–6 months prior to the visit | 0.10521665 | Autism in relatives | 0.08904415 |
| Father’s education before the visit 9–12 years | 0.10168567 | Accidental injuries in relatives | 0.08346737 |
| Anxiolytics dispensation 1–3 months prior to the visit | 0.09413560 | Anxiety disorder 3–5 years prior to the visit | -0.07491725 |
| Accidental injuries 3–6 months prior to the visit | 0.08870387 | Borderline personality disorder 3–6 months prior to the visit | 0.07439240 |
| Antipsychotics dispensation 1 month prior to the visit | 0.08422213 | Anxiety disorder 6–12 months prior to the visit | 0.07389147 |
| Accidental injuries in relatives | 0.08142357 | Borderline personality disorder 1 month prior to the visit | 0.07242722 |
| Major depressive disorder 1–3 months prior to the visit | 0.08128624 | Major depressive disorder 1–3 months prior to the visit | 0.07064241 |
| Accidental injuries 1–3 years prior to the visit | 0.07945228 | Borderline personality disorder at the visit | 0.07028141 |
| Self-harm of undetermined intent 3–6 months prior to the visit | 0.07901935 | Obesity in relatives | 0.06668984 |
| Violent crimal offense ever committed by parents or siblings | 0.07799796 | Major depressive disorder 1–3 years prior to the visit | -0.06662692 |
| Accidental injuries 3–5 years prior to the visit | 0.07730071 | Sarcoidosis in relatives | -0.06346231 |
| Epilepsy 3–5 years prior to the visit | 0.07656211 | Autism 1–3 months prior to the visit | 0.06231805 |
| Borderline personality disorder 3–6 months prior to the visit | 0.07641606 | Intentional self-harm by Blunt object | 0.06138171 |
| Education before the visit 9–12 years | 0.07616503 | Intentional self-harm 3–5 years prior to the visit | 0.06126683 |
| Family income before the visit | 0.07615841 | Anxiety disorder 3–6 months prior to the visit | 0.06070755 |
| Anxiety disorder 3–6 months prior to the visit | 0.07408714 | Anxiolytics dispensation 1 month prior to the visit | 0.05845477 |
| Obesity 1–3 years prior to the visit | 0.07369072 | Antidepressants dispensation 1–3 years prior to the visit | -0.05711181 |
| Autism in relatives | 0.07323221 | Intentional self-harm by Jumping from a high place | 0.05692208 |
| Self-harm of undetermined intent by Poisoning | 0.07316684 | Epilepsy 3–5 years prior to the visit | 0.05499319 |
| Bipolar disorders 1 month prior to the visit | 0.07284746 | Benzodiazepines and related drugs dispensation 3–5 years prior to the visit | -0.05461747 |
| Major depressive disorder 6–12 months prior to the visit | 0.07113840 | Substance use disorder 1–3 months prior to the visit | -0.05444658 |
| Major depressive disorder at the visit | 0.07096589 | Asthma 3–6 months prior to the visit | 0.05443818 |
| Asthma 3–6 months prior to the visit | 0.07030926 | Major depressive disorder 6–12 months prior to the visit | 0.05363446 |
| Mother not receiving sickness or employment injury benefit before the visit | 0.06939357 | Father not receiving income support | 0.05281869 |
| Self-harm of undetermined intent 1–3 months prior to the visit | 0.06925090 | Accidental injuries 1–3 years prior to the visit | 0.04430289 |
| Borderline personality disorder 1–3 months prior to the visit | 0.06881306 | Substance use disorder 1 month prior to the visit | -0.04418758 |
| Schizophrenia 6–12 months prior to the visit | -0.06855096 | Other personality disorders in relatives | 0.04179500 |
| Intentional self-harm by Smoke, fire and flames | 0.06796867 | Intentional self-harm by Other specified means | 0.04139260 |
| Intentional self-harm by Blunt object | 0.06738105 | Anxiolytics dispensation 1–3 months prior to the visit | 0.04016128 |
| Intentional self-harm 3–5 years prior to the visit | 0.06729256 | Substance use disorder 6–12 months prior to the visit | 0.03827530 |
| Benzodiazepines and related drugs dispensation 3–5 years prior to the visit | -0.06610566 | Self-harm of undetermined intent by Poisoning | 0.03814017 |
| Autism 1–3 months prior to the visit | 0.06602308 | Self-harm of undetermined intent 1 month prior to the visit | 0.03798396 |
| Benzodiazepines and related drugs dispensation 3–6 months prior to the visit | 0.06557903 | Anxiety disorder 1–3 months prior to the visit | 0.03733398 |
| Antipsychotics dispensation 1–3 years prior to the visit | -0.06530699 | Intellectual disability 6–12 months prior to the visit | 0.03726633 |
| Accidental injuries 1–3 months prior to the visit | 0.06498268 | Education before the visit ≥12years | -0.03689553 |
| Anxiety disorder 1–3 months prior to the visit | 0.06112544 | Father’s education before the visit ≥12years | -0.03646974 |
| Borderline personality disorder 1 month prior to the visit | 0.06091941 | Mother not receiving sickness or employment injury benefit before the visit | 0.03643556 |
| Epilepsy in relatives | 0.05910716 | Accidental injuries 3–5 years prior to the visit | 0.03605140 |
| Self-harm of undetermined intent 1 month prior to the visit | 0.05889716 | Education before the visit ≤9 years | 0.03584346 |
| Sarcoidosis in relatives | -0.05829986 | Antipsychotics dispensation 1–3 months prior to the visit | 0.03508604 |
| Antipsychotics dispensation 1–3 months prior to the visit | 0.05808717 | Other psychotic disorder 1–3 years prior to the visit | -0.03372133 |
| Accidental injuries 6–12 months prior to the visit | 0.05786485 | Mother’s education before the visit 9–12 years | 0.03335663 |
| Anxiolytics dispensation 1 month prior to the visit | 0.05629680 | Violent crimal offense ever committed by parents or siblings | 0.03205880 |
| Other personality disorders 3–6 months prior to the visit | 0.05617967 | Intentional self-harm by Hanging, strangulation and suffocation | 0.02998441 |
| Conduct disorder in relatives | 0.05475720 | Anxiety disorder 1–3 years prior to the visit | -0.02917960 |
| Substance use disorder 6–12 months prior to the visit | 0.05124594 | Schizophrenia 6–12 months prior to the visit | -0.02724422 |
| Other personality disorders in relatives | 0.05036806 | Other personality disorders 1 month prior to the visit | 0.02679711 |
| Intentional self-harm by Other specified means | 0.04960806 | Anxiolytics dispensation 3–6 months prior to the visit | 0.02641630 |
| Mother being employed before the visit | 0.04878507 | Accidental injuries 1–3 months prior to the visit | 0.02632162 |
| Father being employed before the visit | 0.04873309 | Antiepileptics dispensation 3–6 months prior to the visit | 0.02627058 |
| Attention-deficit/hyperactivity disorder 6–12 months prior to the visit | -0.04590794 | Bipolar disorders 1 month prior to the visit | 0.02563887 |
| Other personality disorders 1 month prior to the visit | 0.04590013 | Self-harm of undetermined intent 1–3 months prior to the visit | 0.02408264 |
| Anxiolytics dispensation 3–6 months prior to the visit | 0.04533812 | Non-violent criminal offense within 1 month prior to the visit | -0.02363987 |
| Father not receiving income support | 0.04465548 | Self-harm of undetermined intent by Unspecified means | 0.02294013 |
| Anxiety disorder 1–3 years prior to the visit | -0.04089838 | Antipsychotics dispensation 1 month prior to the visit | 0.02258658 |
| Other personality disorders at the visit | 0.04065366 | Psychostimulants dispensation 3–5 years prior to the visit | -0.01998549 |
| Non-violent criminal offense within 1 month prior to the visit | -0.03978245 | Other psychotic disorder 3–6 months prior to the visit | 0.01905433 |
| Bipolar disorders 1–3 years prior to the visit | -0.03891412 | Intellectual disability 1 month prior to the visit | 0.01740629 |
| Mother receiving income support | 0.03870964 | Other personality disorders 3–6 months prior to the visit | 0.01692443 |
| Other psychotic disorder 6–12 months prior to the visit | 0.03792804 | Father being employed before the visit | 0.01533143 |
| Antiepileptics dispensation 3–6 months prior to the visit | 0.03724512 | Self-harm of undetermined intent 3–6 months prior to the visit | 0.01459560 |
| Antiepileptics dispensation 6–12 months prior to the visit | -0.03702725 | Education before the visit 9–12 years | 0.01425221 |
| Anxiety disorder 6–12 months prior to the visit | 0.03579137 | Antiepileptics dispensation 1–3 months prior to the visit | 0.01395470 |
| Conduct disorder 1–3 years prior to the visit | 0.03539124 | Epilepsy in relatives | 0.01186502 |
| Antidepressants dispensation 6–12 months prior to the visit | -0.03434679 | Antidepressants dispensation 6–12 months prior to the visit | -0.01070303 |
| Receiving income support before the visit | 0.03376709 | Major depressive disorder at the visit | 0.00999575 |
| Antidepressants dispensation 3–5 years prior to the visit | -0.03369448 | Mood stabilizer dispensation 1–3 years prior to the visit | -0.00715047 |
| Asthma 3–5 years prior to the visit | 0.03346650 | Non-violent criminal offense 1–3 months prior to the visit | -0.00671803 |
| Attention-deficit/hyperactivity disorder 1 month prior to the visit | -0.03242019 | Asthma 6–12 months prior to the visit | 0.00612424 |
| Schizophrenia 3–6 months prior to the visit | -0.03220383 | Bipolar disorders 1–3 years prior to the visit | -0.00334833 |
| Mother not receiving income support | 0.02973708 | Antidepressants dispensation 1–3 months prior to the visit | -0.00026585 |
| Other psychotic disorder 3–5 years prior to the visit | -0.02957893 |  |  |
| Mood stabilizer dispensation 1–3 years prior to the visit | -0.02832784 |  |  |
| Autism 1–3 years prior to the visit | -0.02763642 |  |  |
| Asthma 6–12 months prior to the visit | 0.02758009 |  |  |
| Intentional self-harm by Jumping from a high place | 0.02661579 |  |  |
| Antidepressants dispensation 1–3 years prior to the visit | -0.02068574 |  |  |
| Mother’s education before the visit 9–12 years | 0.02035131 |  |  |
| Mother being unemployed before the visit | 0.01998762 |  |  |
| Attention-deficit/hyperactivity disorder in relatives | -0.01967379 |  |  |
| Non-violent crimal offense ever committed by parents or siblings | 0.01906840 |  |  |
| Major depressive disorder 1–3 years prior to the visit | -0.01730180 |  |  |
| Other psychotic disorder 3–6 months prior to the visit | 0.01450438 |  |  |
| Non-violent criminal offense 1–3 months prior to the visit | -0.01447410 |  |  |
| Attention-deficit/hyperactivity disorder 1–3 years prior to the visit | 0.01418386 |  |  |
| Antiepileptics dispensation 3–5 years prior to the visit | 0.01385876 |  |  |
| Anxiety disorder at the visit | 0.01327854 |  |  |
| Mother’s education before the visit ≥12years | 0.01318510 |  |  |
| Father’s education before the visit ≤9 years | 0.01306089 |  |  |
| Obesity in relatives | 0.01202536 |  |  |
| Intellectual disability 6–12 months prior to the visit | 0.01137160 |  |  |
| Crohn's disease in relatives | 0.00855746 |  |  |
| Self-harm of undetermined intent by Sharp object | 0.00819435 |  |  |
| Being employed before the visit | 0.00750487 |  |  |
| Antiepileptics dispensation 1–3 months prior to the visit | 0.00677155 |  |  |
| Type 1 Diabetes 3–5 years prior to the visit | -0.00657392 |  |  |
| Father not receiving sickness or employment injury benefit before the visit | 0.00651481 |  |  |
| Substance use disorder at the visit | 0.00648474 |  |  |
| Autism at the visit | 0.00478031 |  |  |
| Intellectual disability in relatives | 0.00282057 |  |  |
| Drugs used for addictive disorders dispensation 1 month prior to the visit | -0.00073432 |  |  |
