# Supplementary material for: Predicting suicide attempt or suicide death following a visit to psychiatric specialty care: A machine learning study using Swedish national registry data
Source: PLoS Med. 2020 Nov 6;17(11):e1003416. doi: 10.1371/journal.pmed.1003416 (PMC7647056; doi:10.1371/journal.pmed.1003416)
Supplement: Attachment — Submitted filename: Response to Reviewer (3).pdf [file pmed.1003416.s016.pdf]

Requests from the editors:

1.Response to reviewers: Please fully respond to the comments of reviewer 3, including whether the calibration plot was derived from the training data set or the validation set and in the methods or the legend of Figure 2 please describe how the plots were created/what is represented in the plots.

Response: We have addressed these comments fully (see below).

2.Please revise your title according to PLOS Medicine's style. Your title must be nondeclarative and not a question. It should begin with main concept if possible. Please place the study design ("A randomized controlled trial," "A retrospective study," "A modelling study," etc.) in the subtitle (ie, after a colon). We suggest: "Predicting suicide attempt or death following a visit to psychiatric specialty care: A machine learning study of Swedish national registry data" or similar.

Response: We have revised the title according to PLOS Medicine style, and agree with your suggestion.

"Predicting suicide attempt or death following a visit to psychiatric specialty care: A machine learning study of Swedish national registry data"

3.Abstract: Methods and Findings: If possible please present the p values for both the 30 day and 90 day models

Response: The p value ($p<0.01$) was the same for both models. We have now rephrased the sentence as follows:

"The area under the receiver operating characteristic (ROC) curves (AUCs) on the test set were 0.88 (95% confidence interval [CI]=0.87–0.89) and 0.89 (95% CI=0.88–0.90) for the outcome within 90 days and 30 days, respectively, ***both being*** *significantly better than chance (i.e. AUC = 0.50) (p < 0.01)."*

4.Author Summary: First bullet point under "What do these findings mean?": Please revise to: Our findings suggest that combining machine learning with registry data has potential to accurately predict short -term suicidal behavior.

Response: We have rephrased the first bullet point under "What do these findings mean?" as follows:

"Our findings suggest that *combining machine learning with registry data has the potential to accurately predict short-term suicidal behavior."*

5.Discussion: Middle of paragraph on page 13: Please rephrase the term "completed suicide" in the following sentence; we suggest: "However, it is difficult to directly compare the models from the two studies, given the differences in definition of the predicted outcome (suicide death vs suicidal attempt or death) and time window of interest between the studies." or similar.

Response: To keep suicide terminology consistent throughout the manuscript, we have rephrased as per your suggestion:

*"However, it is difficult to directly compare the models from the two studies, given the differences in definition of the predicted outcome (**suicide death vs. suicide attempt or death**) and time window of interest between the studies."*

6.Figure 1: Please change the colors/patterns of the solid and dotted lines to make them easier to differentiate.

Response: We have been changed the colors of the lines to make them easier to differentiate.

7.Supporting information file: eTable 7: The word "days" is missing from the legend following "90" and "30"

Response: Thank you for spotting the mistakes. Now corrected.

8.TRIPOD Guideline: S1 Checklist is not present in the file inventory, please provide the TRIPOD checklist. When completing the checklist, please use section names and paragraph numbers to refer to locations within the text, rather than page numbers.

Response: We have now uploaded the TRIPOD checklist.

Comments from the reviewers:

Reviewer #3: The authors have done a good job in making clarifications and addressing reviewer concerns. The additional limitations added to the discussion are important.

I am still confused by the calibration plot. I cannot find in the paper if the calibration plot is created using the training data or the validation data set. It should be created in the validation data set using percentile bins from the training data. It appears that deciles were used for the calibration plot (but why are there are only 9 dots instead of 10 ?). I am still very surprised that the observed probability of a suicide attempt in the highest risk group is 100%. The math doesn't make sense here, because if this was created in the validation data set, then there would be about 10,000 visits in the highest risk decile; given the graph it says that nearly 100% of those visits were observed to have a suicide attempt following the visits. that would be about 10,000 suicide attempts, but there should only be about 3,726 suicide attempts in the entire validation data set. The math continues to be a problem with if the calibration plot was created with the training data set.

Please provide more details (not just the function that was used) on how these calibration plots were created.

A common approach is to divide your visits into deciles, these deciles are on the x axis with the mean predicted risk in that percentile. Then on the y-axis is the observed proportion of visits followed by a suicide attempt. At the end of the day a calibration plot needs to indicate in specific bins of people defined by risk, how similar is their predicted risk (from the model) and their observed risk (proportion of those visits with an event following the visit).

Response: Thank you for giving us another opportunity to clarify how the calibration curves were generated.

The calibration curves were derived from the test set. This has been clarified in the Methods section on **page 9 line 5**:

*"The Brier score (equal to zero under perfect calibration), along with calibration plots, was used to assess model calibration **in the test set** (i.e., the agreement between observed proportion of positives and mean predicted risk of the outcome in different risk strata)"*

The number of bins (n_bins) is a parameter of the python function sklean.calibration.calibration_curve (https://scikit-learn.org/stable/modules/generated/sklearn.calibration.calibration_curve.html). It is the number of bins to split the [0, 1] interval and the default is 5 (i.e., predicted risk at 0.0, 0.2, 0.4, 0.6, 0.8, 1.0). The selection of the parameter value is somewhat arbitrary. The parameter does not have to be 10, but can be assigned any positive whole number. A principle guides the choice of the number of bins, which we have followed – the size of subsample in each bin should not be too small. Bins with no subsample, however, would not affect the overall pattern of the calibration curve, because no value would be returned for such bins.

In our study, the parameter (n_bins or number of risk bins) was set to be 9 for the 90-day outcome and 8 for the 30-day outcome. Detailed numbers and calculations underlying the curves are shown in the tables below. To show this as clearly as possible, tables have been combined into one table and added to the online supplement as eTable 9.

In the Results section on **page 10 line 22:**
"… More details can be found in eTable 9."

For the 90-day outcome, all 31 index visits in the risk group with predicted risk between 0.889 and 1.000 were followed by a suicidal event within 90 days. Hence, the observed proportion of positives in the risk group was 100%. For the 30-day outcome, only 6 dots were generated, given no index visits were in the last two bins.

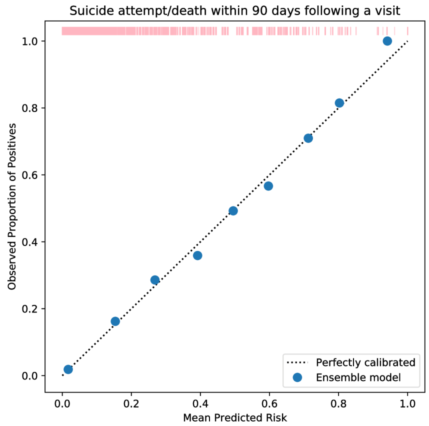

Bin	Range of predicted risk	Number of index visits	Number of true positives	Mean predicted risk (x-axis)	Observed proportion of positives (y-axis)
1	[0.000, 0.111)	100,816	1,858	0.0171	0.0184
2	[0.111, 0.222)	3,966	643	0.1535	0.1621
3	[0.222, 0.333)	1,081	309	0.2684	0.2858
4	[0.333, 0.444)	992	356	0.3918	0.3589
5	[0.444, 0.556)	599	295	0.4950	0.4925
6	[0.556, 0.667)	383	217	0.5969	0.5666
7	[0.667, 0.778)	327	232	0.7124	0.7095
8	[0.778, 0.889)	81	66	0.8026	0.8148
9	[0.889, 1.000]	31	31	0.9415	1.0000

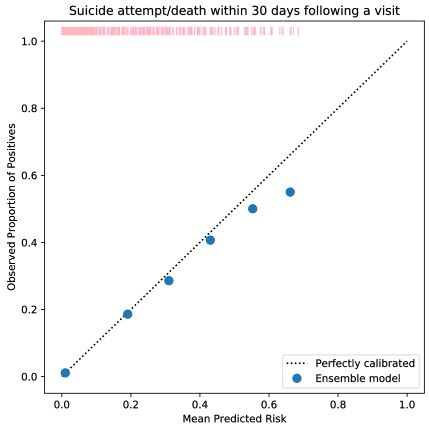

Bin	Range of predicted risk	Number of index visits	Number of true positives	Mean predicted risk (x-axis)	Observed proportion of positives (y-axis)
1	[0.000, 0.125)	105,266	1,130	0.0101	0.0107
2	[0.125, 0.250)	1,614	300	0.1909	0.1859
3	[0.250, 0.375)	837	239	0.3102	0.2855
4	[0.375, 0.500)	423	172	0.4301	0.4066
5	[0.500, 0.625)	96	48	0.5528	0.5000
6	[0.625, 0.750)	40	22	0.6614	0.5500
7	[0.750, 0.875)	0	0	NA	NA
8	[0.875, 1.000]	0	0	NA	NA

We would like to illustrate in this response letter how the calibration curves would look like if the parameter value (i.e., number of risk bins) were set to be 10. For the 90-day outcome, there would be too few index visits (17 and 21) in the 9th and 10th bins. For the 30-day outcome, there would be only 2 index visits in the 8th bin, resulting in a relatively large distortion of the curve. Therefore, we did not set the parameter to be 10. This can be seen in the two calibration plots below for illustrative purposes:

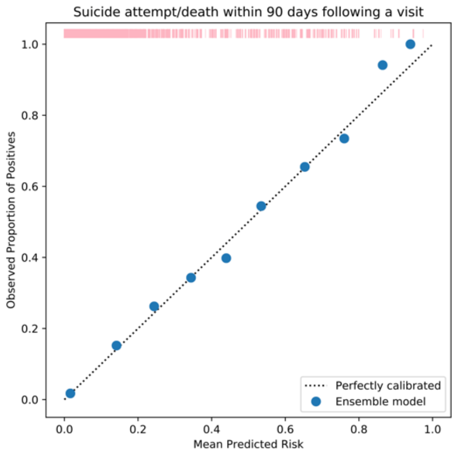

Bin	Range of predicted risks	Number of index visits	Number of true positives	Mean predicted risk (x-axis)	Observed proportion of positives (y-axis)
1	[0.0, 0.1)	99,849	1,725	0.0162	0.0173
2	[0.1, 0.2)	4,683	712	0.1415	0.1520
3	[0.2, 0.3)	1,064	279	0.2438	0.2622
4	[0.3, 0.4)	735	252	0.3440	0.3429
5	[0.4, 0.5)	837	333	0.4396	0.3978
6	[0.5, 0.6)	450	245	0.5346	0.5444
7	[0.6, 0.7)	394	258	0.6531	0.6548
8	[0.7, 0.8)	226	166	0.7602	0.7345
9	[0.8, 0.9)	17	16	0.8644	0.9412
10	[0.9, 1.0]	21	21	0.9399	1.0000

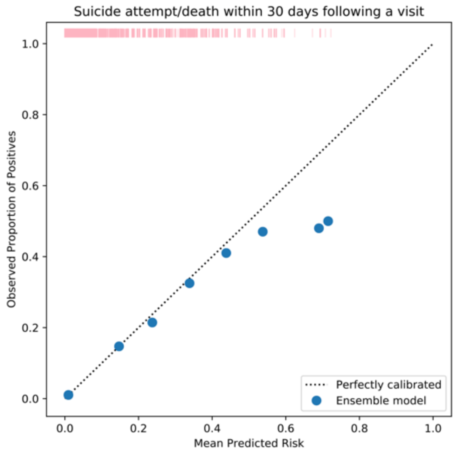

Bin	Range of predicted risks	Number of index visits	Number of true positives	Mean predicted risk (x-axis)	Observed proportion of positives (y-axis)
1	[0.0, 0.1)	104,715	1,076	0.0096	0.0103
2	[0.1, 0.2)	1,494	220	0.1471	0.1473
3	[0.2, 0.3)	970	208	0.2374	0.2144
4	[0.3, 0.4)	646	210	0.3385	0.3251
5	[0.4, 0.5)	256	105	0.4382	0.4102
6	[0.5, 0.6)	168	79	0.5374	0.4702
7	[0.6, 0.7)	25	12	0.6900	0.4800
8	[0.7, 0.8)	2	1	0.7150	0.5000
9	[0.8, 0.9)	0	0	NA	NA
10	[0.9, 1.0]	0	0	NA	NA
