# Supplementary material for: Predicting suicide attempt or suicide death following a visit to psychiatric specialty care: A machine learning study using Swedish national registry data
Source: PLoS Med. 2020 Nov 6;17(11):e1003416. doi: 10.1371/journal.pmed.1003416 (PMC7647056; doi:10.1371/journal.pmed.1003416)
Supplement: Attachment — Submitted filename: Review_plos one medicine.docx [file pmed.1003416.s013.docx]

This is a well-written manuscript, analyzing big data on suicide attempt/death to develop prediction models based on several machine learning algorithms and describing its methodology in detail. Particularly, models developed in this study showed relatively higher PPV despite very low prevalence of suicide outcome, compared with other machine learning studies. My comments are as follow.

1. In this study, suicide attempt was defined as intentional self-harm (ICD-10: X60–X84) or self-harm of undetermined intent (ICD-10: Y10-Y34). Considering that the study population were young adults, a considerable portion of self-harm events might correspond to non-suicidal self-injury. It is controversial whether non-suicidal self-injury represents actual suicide risk. Although the authors already mentioned self-harm behavior in the discussion section as limitation, non-suicidal self-injury should be addressed to clarify suicide outcome more clearly.

2. Prediction models based on machine learning algorithms may be useful in screening at risk population. But, models developed in this study had archived relatively low sensitivity despite high specificity and good AUC performance.
