# Supplementary material for: Predicting suicide attempt or suicide death following a visit to psychiatric specialty care: A machine learning study using Swedish national registry data
Source: PLoS Med. 2020 Nov 6;17(11):e1003416. doi: 10.1371/journal.pmed.1003416 (PMC7647056; doi:10.1371/journal.pmed.1003416)
Supplement: Attachment — Submitted filename: Suicide risk prediction review.docx [file pmed.1003416.s014.docx]

This is an interesting manuscript using an ensemble approach to predict suicide attempt and death following a health care visits. While this could be a good addition to the literature, some more information on the data sources, methods used, and results should be provided. In particular, code implementing the methods should be included and ideally the models learned should be published with the paper so others can evaluate the model in their health system for potential use. As the paper is right now, very little of the work is reproducible and the reported results are noticeably higher than previously published work in this area.

Detailed comments

Description of data sources

- More characteristics should be reported in Table 1. In particular, I would like to see what proportion of visits are outpatient, ED visit, and in-patient. It would be helpful to see some information on diagnosis as well so the audience has some idea on what type of population this sample represents.
- Do the authors have any information the positive predicted value of their outcome in this sample via chart review? What proportion of events identified with their codes are true events when chart reviewed?
- I am specifically concerned about the positive predicted value of events that occur after an in-patient or emergency department visit. Do the authors have any data on the percentage of those events that are true new events as opposed to follow-up visits for the event that caused the ED or inpatient stay?

Description of statistical methods

- The parameter values for each model that were searched over in model training need to be reported.
- Code should be made available to implement their analysis.
- The trained models themselves so that other researchers could evaluate how these models work in their health system. The AUC for these models is very high. It is important that these models can be evaluated in other settings.
- I would like to see the performance of the models separately for outpatient and inpatient/ED visits. Specifically relating to my concern about follow-up visits potentially being counted as new events for the inpatient/ED visits.
- It is unclear to me which variable were included / excluded in the model that use EMR-based data, and what extra data is included in the bigger model for which the main results of the paper refer to.

Results

- The positive predicted values reported seems quite high and does not appear correct based on other information reported in the paper. Based on Table 3 the positive predicted value of the 95^th^ percentile for the 90-day outcome should be 35% but in Table 2 it is reported as 51%
- In all figures what model is being described should be indicated in the caption.
- It is unclear how the ensemble methods are put together. The weights associated with each model in the ensemble model combining all 4 top-performing models should be reported. Based on the results, it looks to me like the ensemble results are mostly driven by the gradient boosting model.
- I would like to see the number of people and number of visits in each of the risk thresholds. I find it very surprising that the risk of suicide attempt, both predicted and observed is nearly 1 for anyone in the sample. Please indicate in the caption of the plot what the dots are in the calibration plot.
