# Supplementary material for: Predicting suicide attempt or suicide death following a visit to psychiatric specialty care: A machine learning study using Swedish national registry data
Source: PLoS Med. 2020 Nov 6;17(11):e1003416. doi: 10.1371/journal.pmed.1003416 (PMC7647056; doi:10.1371/journal.pmed.1003416)
Supplement: Attachment — Submitted filename: Response_to_reviewers.docx [file pmed.1003416.s015.docx]

We would like to thank the editor and the reviewer for giving us the opportunity to revise and resubmit our manuscript entitled “Predicting suicide attempt/death following a visit to psychiatric specialty care by applying machine learning to the Swedish national registry data” (PMEDICINE-D-20-00084R1). We have now carefully revised the manuscript according to the insightful comments listed below. We think that the quality and readability of the manuscript have been considerably improved as a consequence of these changes.

Requests from the editors:

Specific point – You will note that is repeatedly raised among referees that the study is not reproducible and that without the code and parameters for others to replicate this study is severely limited. We are unable to consider a resubmission unless the code is placed in a repository such as Github and all parameters and associated data made freely available. This is regardless of other revisions undertaken.

**Reply:** Thank you for clarifying this. We have now placed the code in the online repository, Github (https://github.com/qichense/suicide_ml/). As for data availability, we have included all tabular data in the paper and appendices. However, in accordance with the Swedish Public Access to Information and Secrecy Act, the Swedish national register data contains sensitive personal information and thus cannot be made publicly available. Data used in the current study can be requested by contacting the Swedish National Board of Health and Welfare ([registerservice@socialstyrelsen.se](mailto:registerservice@socialstyrelsen.se)) and Statistics Sweden ([Mikrodata.individ@scb.se](mailto:Mikrodata.individ@scb.se)). We have taken this approach in previous register-based publication from our group in PLoS Medicine (1), which is a similar approach to another recent publication using the Swedish registry data (2).

General points:

Abstract – please add some demographic information to the abstract as well as mean ages of male and female; please add p values where 95% CIs are given; please provide a sentence on the limitations of the study as the final sentence of the ‘Methods and Findings’ section; ‘models outperforming existing models in predicting relatively short-term suicide attempt/death.’ (also page 10) – unless you perform side-by-side comparisons and show superiority you need to remove such statements. Any comparisons added in revision would be assessed by referees.

**Reply:** We have revised the abstract accordingly.

The revised abstract reads as follows:

*“Methods and findings*

*The study sample consisted of 541,300 inpatient and outpatient visits by 126,205 patients aged 18 to 39 to psychiatric specialty care in Sweden between Jan. 1, 2011 and Dec. 31, 2012.* ***The mean (SD) age of males was 27.7 (6.2) years, while the mean (SD) age of females was 27.3 (6.3) years.*** *A total of 425 candidate predictors covering demographic characteristics, socioeconomic status, electronic medical records, criminality, as well as family history of disease and crime, were extracted from the Swedish registry data. The sample was randomly split into an 80% training set containing 433,024 visits, and a 20% test set containing 108,276 visits. Models were trained separately for suicide attempt/death within 90 and 30 days following a visit using multiple machine learning algorithms. Model discrimination and calibration were both evaluated. Among all eligible visits, 3.5% (18,682) were followed by a suicide attempt/death within 90 days and 1.7% (9,099) within 30 days. The final models were based on ensemble learning that combined predictions from elastic net penalized logistic regression, random forest, gradient boosting, and a neural network. The area under the receiver operating characteristic (ROC) curves (AUCs) on the test set were 0.88 (95% confidence interval [CI]=0.87–0.89) and 0.89 (95% CI=0.88–0.90) for the outcome within 90 days and 30 days, respectively,* ***significantly better than chance (AUC=0.5) (p < 0.01)****.* ***Sensitivity, specificity, and predictive values were reported at different risk thresholds****.* ***The generalizability of the derived models to other populations, however, is yet to be tested.***

*Conclusions*

*By combining the ensemble method of multiple machine learning algorithms and high quality data solely from the Swedish registers,* ***we developed prognostic models to predict short-term suicide attempts and death with good discrimination and calibration****. Whether novel predictors can improve predictive performance requires further investigation.”*

Data – I would remove this ‘they are subject to secrecy I’ and on resubmission state where code can be accessed and this needs to be live for reviewers to access.

**Reply:** We have removed that phrase. We now write: “In accordance with the Swedish Public Access to Information and Secrecy Act, the Swedish national register data contains sensitive personal information and thus cannot be made publicly available. Data used in the current study can be requested by contacting the Swedish National Board of Health and Welfare ([registerservice@socialstyrelsen.se](mailto:registerservice@socialstyrelsen.se)) and Statistics Sweden ([Mikrodata.individ@scb.se)](mailto:Mikrodata.individ@scb.se)). The code has been placed in a repository on Github (https://github.com/qichense/suicide_ml/).”

At this stage, we ask that you include a short, non-technical Author Summary of your research to make findings accessible to a wide audience that includes both scientists and non-scientists. The Author Summary should immediately follow the Abstract in your revised manuscript. This text is subject to editorial change and should be distinct from the scientific abstract.

**Reply:** We have included the following short, non-technical summary:

Author Summary

Why was this study done?

- Suicidal behavior is overrepresented in people with mental illness and contributes to the substantial public health burden of psychiatric conditions. Accurately predicting suicidal behavior has long been challenging.
- The potential of applying machine learning to linked national datasets to predict suicidal behavior remains unknown.

What did the researchers do and find?

- We identified a sample of 541,300 inpatient and outpatient visits to psychiatric specialty care in Sweden during 2011 and 2012. The sample was then divided into a training dataset and a test dataset.
- We first trained prediction models separately for suicide attempt/death within 90-days and 30-days following a visit to psychiatric specialty care, using four different machine learning algorithms. We then used an ensemble method to combine the performance of the trained models with the intention to achieve an overall performance superior than each individual model.
- The final model based on the ensemble method achieved the best predictive performance. This model was applied to test dataset and showed good model discrimination and calibration for both the 90-day and 30-day outcomes.

What do these findings mean?

- Combining machine learning with registry data has potential to accurately predict short-term suicidal behavior.
- An approach combining four machine learning methods showed an overall predictive performance slightly better than each individual model.

Page 7 – please remove the URL and simply say the name of the test and provide the reference.

**Reply:** We have removed the URL and provided the reference.

Comments from the reviewers:

Reviewer #1: This is a well-written manuscript, analyzing big data on suicide attempt/death to develop prediction models based on several machine learning algorithms and describing its methodology in detail. Particularly, models developed in this study showed relatively higher PPV despite very low prevalence of suicide outcome, compared with other machine learning studies. My comments are as follow.

1. In this study, suicide attempt was defined as intentional self-harm (ICD-10: X60–X84) or self-harm of undetermined intent (ICD-10: Y10-Y34). Considering that the study population were young adults, a considerable portion of self-harm events might correspond to non-suicidal self-injury. It is controversial whether non-suicidal self-injury represents actual suicide risk. Although the authors already mentioned self-harm behavior in the discussion section as limitation, non-suicidal self-injury should be addressed to clarify suicide outcome more clearly.

**Reply:** This is a helpful comment, and we note that the distinction between suicidal and non-suicidal self-injury remains controversial, with leading researchers on both sides of the fence. For example, most European and Australasian researchers do not adopt this typology, which is reflected in ICD-10 that does not separate out suicidal and non-suicidal self-injury. At the same time, this is an important issue to consider, and we have added discussion of limitations of the outcome definition and the implication for the findings **on page 15 line 5:**

*“Our definition of suicidal behavior was based on ICD-10 criteria, which does not make distinction between suicidal and non-suicidal self-harm. Although this definition has been widely used in prior research, whether non-suicidal self-harm represents actual suicide risk remains controversial. A certain amount of non-suicidal self-harm events were labeled as positive outcome events, which may have led to an overestimation of PPVs.”*

2. Prediction models based on machine learning algorithms may be useful in screening at risk population. But, models developed in this study had archived relatively low sensitivity despite high specificity and good AUC performance.

**Reply:** We agree with this reviewer that the use of such algorithms may be limited to screening due to their low sensitivities, and have further discussed this in the paper. At the same time, there is no agreement on clinically useful values of the model performance metrics with regard to the research topic, which will depend on how any model is used and what interventions are available. In our model, the metrics varied across risk threshold. For instance, at the 80^th^ percentile risk threshold, the sensitivity is estimated to be 77% for the 90-day outcome and 81% for the 30-day outcome (Table 2). These estimates are relatively high, indicating that the models could play a role in preventing suicidal behavior when effective and non-harmful intervention strategies are linked to the use of any such tool.

So that readers do not focus on sensitivity, specificity, and predictive values solely at the 95^th^ percentile risk threshold in the abstract, we have replaced the sentence below by *“Sensitivity, specificity, and predictive values were reported at different risk thresholds.”*

*~~“At the 95th percentile risk threshold, the respective sensitivities were 47.2% and 52.9%, the specificities were 96.6% and 95.9%, the positive predictive values (PPVs) were 34.9% and 18.7%, and the negative predictive values (NPVs) were 97.9% and 99.1%.”~~*

Reviewer #2

This paper reports the development of algorithms to predict suicide-related outcomes using data from a large registry. The approaches taken appear sound to me, and the contribution to knowledge is important, given the relatively high predictive performance achieved compared to other initiatives. The authors are appropriately aware of the limitations here, in particular the need for cross-site algorithm replication and the distance between the data resources used here and real-world health record capabilities. My comments are minor.

1. The section in the Results on predictive potential of electronic medical records alone felt a little under-described (e.g. it would have been helpful to have the standard list of performance metrics rather than just the AUCs), and the final sentence on p9 looks unfinished. This is an important section because it is likely to represent the element that might most readily be replicated elsewhere, so it would be helpful to have more detail.

**Reply:** This is a helpful comment, and we agree that the section on using data from electronic medical records could be further developed. We have listed additional performance metrics in **eTable 8** and revised the text **on page 11 line 12:**

*“When the candidate predictors were restricted to sex, age at the visit, and those identified from the National Patient Register as well as the Prescribed Drug Register (eTable 1), the AUCs for predicting the outcome events within 90 and 30 days were 0.86 (95% CI: 0.86–0.87) and 0.88 (95% CI: 0.87–0.88), respectively. Compared with the main models, these decreases in AUCs were statistically significant (p<0.05). eTable 8 shows the sensitivity, specificity, and predictive values at different risk thresholds.”*

eTable 8. Model performance metrics at various risk thresholds for predicting suicide attempt/death within 90 and 30 days following a visit to psychiatric specialty care during 2011–2012, **predictors being restricted to sex, age at the visit, and those identified from the National Patient Register as well as the Prescribed Drug Register.**

| **Risk threshold** | **Sensitivity (%)** | **Specificity (%)** | **PPV (%)** | **NPV (%)** |
| --- | --- | --- | --- | --- |
| *Suicide attempt/death within 90 days following a visit* | | | | |
| 99.5^th^ | 9.8 | 99.9 | 72.0 | 96.6 |
| 99^th^ | 17.3 | 99.6 | 64.1 | 96.9 |
| 98^th^ | 27.8 | 99.0 | 51.3 | 97.3 |
| 97^th^ | 36.1 | 98.3 | 44.5 | 97.6 |
| 96^th^ | 41.7 | 97.4 | 38.6 | 97.8 |
| 95^th^ | 46.6 | 96.6 | 34.5 | 97.9 |
| 90^th^ | 60.9 | 92.0 | 22.6 | 98.4 |
| 85^th^ | 69.6 | 87.1 | 17.2 | 98.7 |
| 80^th^ | 75.6 | 82.1 | 14.0 | 98.9 |
| 70^th^ | 82.7 | 72.0 | 10.2 | 99.1 |
| 60^th^ | 87.7 | 61.8 | 8.1 | 99.2 |
| 50^th^ | 91.5 | 51.6 | 6.8 | 99.4 |
| *Suicide attempt/death within 30 days following a visit* | | | | |
| 99.5^th^ | 12.7 | 99.7 | 44.6 | 98.5 |
| 99^th^ | 21.3 | 99.4 | 37.6 | 98.6 |
| 98^th^ | 34.4 | 98.6 | 30.4 | 98.8 |
| 97^th^ | 41.9 | 97.7 | 24.7 | 98.9 |
| 96^th^ | 47.8 | 96.8 | 21.1 | 99.0 |
| 95^th^ | 51.5 | 95.8 | 18.2 | 99.1 |
| 90^th^ | 65.9 | 91.0 | 11.6 | 99.3 |
| 85^th^ | 73.7 | 86.1 | 8.7 | 99.5 |
| 80^th^ | 78.8 | 81.1 | 7.0 | 99.5 |
| 70^th^ | 85.3 | 71.0 | 5.0 | 99.6 |
| 60^th^ | 89.7 | 60.9 | 4.0 | 99.7 |
| 50^th^ | 93.0 | 50.8 | 3.3 | 99.8 |
| PPV: Positive predictive value; NPV: Negative predictive value | | | |  |

2. I think it might be helpful if the text in the 3rd paragraph of the discussion was re-ordered so that the sensitivity and PPVs (and possibly NPVs) of the 95th and 80th percentile alternatives were more explicitly linked and discussed together, as these are most reflective of potential clinical applicability.

**Reply:** We have revised the text as follows **on page 12 line 3**:

*“ … In the current study, at the 95th percentile risk threshold, the model would correctly identify approximately half of all suicide attempts/deaths* *within 90 days (sensitivity 47.2%); among those psychiatric visits that were predicted to be at high risk, around one third were actually followed by suicidal events within 90 days (i.e. PPV 34.7%). Although a higher sensitivity could be achieved at a lower threshold, this would be at the cost of a reduced PPV. At the 80th percentile risk threshold, nearly 80% of all suicide attempts/deaths within 90 days would be correctly identified (sensitivity 77.4%); among those visits that were predicted to be at high risk, one in seven was followed by suicidal events within 90 days (PPV 14.3%). Meanwhile, most visits without suicidal events within 90 days would be correctly identified given the high specificity estimates (96.6% and 82.2% at the 95th and 80th percentile risk thresholds, respectively), while most visits predicted to be low risk were not followed by suicidal events given the high NPV (97.9 % and 99.0% at the 95th and 80th percentile risk thresholds, respectively). Selecting a reasonable risk threshold for predictive values depends on the resources available for subsequent intervention strategies and their implications for individuals and services. When effective intervention strategies are taken into consideration, model performance metrics at other risk thresholds may also be informative. Future research on cost-effective interventions with negligible or no adverse effects are warranted (e.g., increased frequency of follow-up), as such interventions could be allocated to false-positive patients using models with low PPVs. …”*

3. On p11, the statement about NPV being more useful for directing resources is a little problematic, as the consideration here is just focused on a single, albeit important, outcome. In order for NPV to be used in this way, it would have to be established that patients falling into the negative group were at lower risk of all other adverse outcomes; otherwise they might receive suboptimal care just on the grounds of lower suicidal risk.

**Reply:** This is a thoughtful comment, and we have considered it carefully. We agree with the reviewer that screening out on the basis of a high NPV will only be relevant for this specific outcome, and there is a possibility that these people who have been screened out may be at higher risk for other adverse outcomes. We think that this warrants some discussion, and have added the following to the discussion **on page 13 line 7**:

*“Moreover, screening out on the basis of high NPVs will be limited to the outcome of interest, and will not be appropriate if those individuals are at increased risk of other adverse outcomes (e.g., accidents). This suggests that if tools are used in this way, clinicians need to consider risks for other outcomes before these individuals are not further assessed or considered for treatment.”*

4. Although I can understand the reason for restrictions on data access, it would be helpful to have a more explicit statement from the authors about what they intend to do with their algorithms. Clearly there is a need for replication here, but I don't believe that this is going to be possible from the information provided.

**Reply:** The code has been placed in a repository on Github (https://github.com/qichense/suicide_ml/).

In accordance with the Swedish Public Access to Information and Secrecy Act, the Swedish national register data contains sensitive personal information and thus cannot be made publicly available. Data used in the current study can be requested by contacting the Swedish National Board of Health and Welfare ([registerservice@socialstyrelsen.se](mailto:registerservice@socialstyrelsen.se)) and Statistics Sweden ([Mikrodata.individ@scb.se](mailto:Mikrodata.individ@scb.se))

Reviewer #3: This is an interesting manuscript using an ensemble approach to predict suicide attempt and death following a health care visits. While this could be a good addition to the literature, some more information on the data sources, methods used, and results should be provided. In particular, code implementing the methods should be included and ideally the models learned should be published with the paper so others can evaluate the model in their health system for potential use. As the paper is right now, very little of the work is reproducible and the reported results are noticeably higher than previously published work in this area.

**Reply:** The code has been placed in a repository on Github (https://github.com/qichense/suicide_ml/).

Detailed comments

1. Description of data sources

More characteristics should be reported in Table 1. In particular, I would like to see what proportion of visits are outpatient, ED visit, and in-patient. It would be helpful to see some information on diagnosis as well so the audience has some idea on what type of population this sample represents.

**Reply:** We agree that this information is important for the readers to determine whether the generalizability of the included patients. We have added information on outpatient and inpatient proportion (although information on whether or not an in-/outpatient visit was referred from ED is not available in our data sources) as well as information on primary diagnoses at each visit, including substance use disorders, schizophrenia spectrum disorders, bipolar disorder, major depressive disorder, anxiety disorders, borderline personality disorder, attention-deficit/hyperactivity disorder, and others (Table 1).

**Table1. Baseline characteristics of 541,300 eligible visits by 126,205 patients to psychiatric specialty care during 2011–2012**

| **Characteristic** | **Training set**  **n (%)** | **Test set**  **n (%)** |
| --- | --- | --- |
| Visits | 433,024 | 108,276 |
| **Inpatient** | **49,077 (11.3)** | **12,293 (11.4)** |
| **Outpatient** | **383,947 (88.7)** | **95,983 (88.6)** |
| Unique patients | 100,964 | 25,241 |
| Female | 242,944 (56.1) | 62,355 (57.6) |
| Mean (SD) age at the visit, years | 27.3 (6.1) | 27.2 (6.1) |
| **Primary diagnosis** |  |  |
| **Substance use disorders** | **59,178 (13.7)** | **14,427 (13.3)** |
| **Schizophrenia-spectrum disorders** | **27,467 (6.3)** | **6,073 (5.6)** |
| **Bipolar disorder** | **33,005 (7.6)** | **8,412 (7.8)** |
| **Major depressive disorder** | **72,876 (16.8)** | **18,676 (17.2)** |
| **Anxiety disorders** | **86,246 (19.9)** | **21,933 (20.3)** |
| **Borderline personality disorder** | **17,248 (4.0)** | **4626 (4.3)** |
| **Attention-deficit/hyperactivity disorder** | **53,048 (12.3)** | **12,991 (12.0)** |
| **Autism** | **20,831 (4.8)** | **5,170 (4.8)** |
| **Others** | **63,125 (14.6)** | **15,968 (14.7)** |
| Visits followed by |  |  |
| Suicide attempt/death within 90 days ^a^ | 14,675 (3.4) | 4007 (3.7) |
| Intentional self-harm | 13,308 (3.1) | 3696 (3.4) |
| Self-harm with undetermined intent | 1277 (0.3) | 353 (0.3) |
| Death from intentional self-harm | 379 (0.1) | 56 (0.1) |
| Death from self-harm of undetermined intent | 164 (0.04) | 39 (0.04) |
| Suicide attempt/death within 30 days ^a^ | 7188 (1.7) | 1911 (1.8) |
| Intentional self-harm | 6596 (1.5) | 1775 (1.6) |
| Self-harm with undetermined intent | 505 (0.1) | 142 (0.1) |
| Death from intentional self-harm | 144 (0.03) | 19 (0.02) |
| Death from self-harm of undetermined intent | 55 (0.01) | 14 (0.01) |

^a^ Different types of events may occur during the same outcome window

2. Do the authors have any information the positive predicted value of their outcome in this sample via chart review? What proportion of events identified with their codes are true events when chart reviewed?

**Reply:** We do not have information on the positive predictive value via chart review. Nevertheless, according to an external review on the validity of diagnoses in the Swedish national inpatient register (3), the PPVs of the register-based diagnostic codes varied between 85% to 95%. Notably, the PPV of injury, including both accidental injury and self-injury, was 95%. On the basis of this, we think that the positive predictive value of our outcome is likely to be high, despite a lack of specific validation study.

We have acknowledged the limitation in the discussion **on page 15 line 1**:

*“Our outcomes have limited validation. Nevertheless, according to an external review on the validity of diagnoses in the Swedish national inpatient register, the PPVs of the register-based diagnostic codes varied between 85% to 95%. Notably, the PPV of injury, including both accidental injury and self-injury, was 95%. On the basis of this, we think that the positive predictive value of our outcome is likely to be high, despite a lack of specific validation study.”*

3. I am specifically concerned about the positive predicted value of events that occur after an in-patient or emergency department visit. Do the authors have any data on the percentage of those events that are true new events as opposed to follow-up visits for the event that caused the ED or inpatient stay?

**Reply:** This is an important issue to clarify. In fact, we only selected unplanned visits as outcome events as these are assumed to be new incident events. Planned visits were likely to be follow-up healthcare appointments following an incident suicide attempt and were not included.

In the section of outcome definition **on page 6 line 25**, we state:

*“Only unplanned inpatient or outpatient visits with a recorded self-harm were labelled as incident suicide attempts. Planned visits were likely to be follow-up healthcare appointments following an incident suicide attempt and thus were not included.”*

4. Description of statistical methods.

The parameter values for each model that were searched over in model training need to be reported.

**Reply:** We have provided the information in eTable 6 and added the following text **on page 9 line 27**:

*“eTable 6 lists the optimized hyperparameters for each individual model.”*

5. Code should be made available to implement their analysis.

**Reply:** We agree and the code has been placed in a repository on Github (https://github.com/qichense/suicide_ml/).

6. The trained models themselves so that other researchers could evaluate how these models work in their health system. The AUC for these models is very high. It is important that these models can be evaluated in other settings.

**Reply:** The code has been placed in a repository on Github (https://github.com/qichense/suicide_ml/).

7. I would like to see the performance of the models separately for outpatient and inpatient/ED visits. Specifically relating to my concern about follow-up visits potentially being counted as new events for the inpatient/ED visits.

**Reply:** We agree with the reviewer on the relevance of separately investigating model performance for inpatients and outpatients. In fact, we are planning another study where models will be trained separately for inpatient and outpatient visits, as well as subgroups of patients with different diagnoses. We therefore will not further expand the results of the current study.

As clarified above, only unplanned inpatient or outpatient visits with a recorded self-harm were labelled as incident suicide attempts. Planned visits were likely to be follow-up healthcare appointments following an incident suicide attempt and were not included.

8. It is unclear to me which variable were included / excluded in the model that use EMR-based data, and what extra data is included in the bigger model for which the main results of the paper refer to.

**Reply:** We are grateful for this comment, and have further developed the last paragraph of the results section **on page 11 line 12** as follows:

*“When the candidate predictors were restricted to* ***sex, age at the visit, and*** ***those identified from the National Patient Register as well as the Prescribed Drug Register*** ***(eTable 1)****, the AUCs for predicting the outcome events within 90 and 30 days were 0.86 (95% CI: 0.86–0.87) and 0.88 (95% CI: 0.87–0.88), respectively. Compared with the main models, the decrease in AUCs were statistically significant (p<0.05). eTable 8 shows the sensitivity, specificity, and predictive values at different risk thresholds.”*

Variables used in the EMR-based data are listed in the Table below (one part of eTable 1).

| **The Medical Birth Register (1 predictor)** | |
| --- | --- |
| Sex (1 predictor) | |
| **The National Patient Register (299 predictors)** | |
| Age at the visit (1 predictor) | |
| Inpatient/Outpatient visit (1 predictor) | |
| Planned/unplanned visit (2 dummy indicators) | Missing (0.57%) |
| Hospitalization within 1 month prior to the visit (1 predictor) | |
| Hospitalization 1–3 months prior to the visit (1 predictor) | |
| Unplanned visit within 1 month prior to the visit (1 predictor) | |
| Unplanned visit 1–3 months prior to the visit (1 predictor) | |
| Diagnosis at the visit (37 predictors) | Intentional self-harm, Self-harm of undetermined intent, Attention-deficit/hyperactivity disorder, Substance use disorder, Intellectual disability, Autism, Anxiety disorder, Major depressive disorder, Bipolar disorders, Schizophrenia, Other psychotic disorder, Conduct disorder, Oppositional defiant disorder, Antisocial personality disorder, Borderline personality disorder, Other personality disorders, Obesity, Epilepsy, Asthma, Eczema, Hypertension, Type 1 Diabetes, Type 2 Diabetes, Parkinson disease/Parkinsonism, Ankylosing spondylitis, Celiac disease, Crohn's disease, Grave's disease, Hashimoto's disease, Multiple sclerosis, Psoriasis, Rheumatoid arthritis, Sarcoidosis, Sjogren's syndrome, Systemic lupus erythematosus, Ulcerative colitis, Accidental injuries |
| Diagnosis 1 month prior to the visit (37 predictors) |  |
| Diagnosis 1–3 months prior to the visit (37 predictors) |  |
| Diagnosis 3–6 months prior to the visit (37 predictors) |  |
| Diagnosis 6–12 months prior to the visit (37 predictors) |  |
| Diagnosis 1–3 years prior to the visit (37 predictors) |  |
| Diagnosis 3–5 years prior to the visit (37 predictors) |  |
| Method used in prior suicide attempt (32 predictors) | Poisoning, Hanging, strangulation and suffocation, Drowning and submersion, Handgun discharge, Rifle, shotgun and larger firearm discharge, Other and unspecified firearm discharge, Explosive material, Smoke, fire and flames, Steam, hot vapours and hot objects, Sharp object, Blunt object, Jumping from a high place, Jumping or lying before moving object, Crashing of motor vehicle, Other specified means, Unspecified means  Self-harm of undetermined intent by Poisoning, Hanging, strangulation and suffocation, Drowning and submersion, Handgun discharge, Rifle, shotgun and larger firearm discharge, Other and unspecified firearm discharge, Explosive material, Smoke, fire and flames, Steam, hot vapours and hot objects, Sharp object, Blunt object, Jumping from a high place, Jumping or lying before moving object, Crashing of motor vehicle, Other specified means, Unspecified means |
| **The Prescribed Drug Register (48 predictors)** | |
| Medication dispensation 1 month prior to the visit (8 predictors) | Antipsychotics, Antidepressants, Anxiolytics, Mood stabilizer, Psychostimulants, Drugs used for addictive disorders, Benzodiazepines and related drugs, Antiepileptics |
| Medication dispensation 1–3 months prior to the visit (8 predictors) |  |
| Medication dispensation 3–6 months prior to the visit (8 predictors) |  |
| Medication dispensation 6–12 months prior to the visit (8 predictors) |  |
| Medication dispensation 1–3 years prior to the visit (8 predictors) |  |
| Medication dispensation 3–5 years prior to the visit (8 predictors) |  |

Extra data included in the bigger model are listed in the Table below (the other part of eTable1)

| **The National Crime Register (8 predictors)** | |
| --- | --- |
| Criminal offense within 1 month prior to the visit (2 predictors) | Violent criminal offense, Non-violent criminal offense |
| Criminal offense 1–3 months prior to the visit (2 predictors) |  |
| Criminal offense 3–6 months prior to the visit (2 predictors) |  |
| Criminal offense 6–12 months prior to the visit (2 predictors) |  |
| **The Longitudinal integration database for health insurance and labor market studies (8 predictors)** | |
| Education before the visit (3 dummy indicators) | ≤9 years, 9–12 years, ≥12 years, missing (2.45%) |
| Being employed before the visit (1 predictor) | |
| Receiving study income before the visit (1 predictor) | |
| Receiving sickness or employment injury benefit before the visit (1 predictor) | |
| Receiving income support before the visit (1 predictor) | |
| Family income before the visit (1 predictor) | |
| **The Multi-generation Register + The National Patient Register (39 predictors)** | |
| Family history of diseases (39 predictors) | Intentional self-harm, Self-harm of undetermined intent, Death due to intentional self-harm, Death due to self-harm of undetermined intent, Attention-deficit/hyperactivity disorder, Substance use disorder, Intellectual disability, Autism, Anxiety disorder, Major depressive disorder, Bipolar disorders, Schizophrenia, Other psychotic disorder, Conduct disorder, Oppositional defiant disorder, Antisocial personality disorder, Borderline personality disorder, Other personality disorders, Obesity, Epilepsy, Asthma, Eczema, Hypertension, Type 1 Diabetes, Type 2 Diabetes, Parkinson disease/Parkinsonism, Ankylosing spondylitis, Celiac disease, Crohn's disease, Grave's disease, Hashimoto's disease, Multiple sclerosis, Psoriasis, Rheumatoid arthritis, Sarcoidosis, Sjogren's syndrome, Systemic lupus erythematosus, Ulcerative colitis, Accidental injuries, |
| **The Multi-generation Register + The National Crime Register (4 predictors)** | |
| Crime ever committed by parents or siblings (2 predictors) | Violent criminal offense, Non-violent criminal offense |
| Crime committed by parents or siblings within 1 year before the visit (2 predictors) |  |
| **The Multi-generation Register + The Longitudinal integration database for health insurance and labor market studies (18 predictors)** | |
| Mother’s education before the visit (3 dummy indicators) | ≤9 years, 9–12 years, ≥12 years, missing (5.27%) |
| Mother being employed before the visit (2 dummy indicators) | Missing (5.15%) |
| Mother receiving sickness or employment injury benefit before the visit (2 dummy indicators) | Missing (5.15%) |
| Mother receiving income support (2 dummy indicators) | Missing (5.15%) |
| Father’s education before the visit (3 dummy indicators) | ≤9 years, 9–12 years, ≥12 years, missing (12.48%) |
| Father being employed before the visit (2 dummy indicators) | Missing (12.24%) |
| Father receiving sickness or employment injury benefit before the visit (2 dummy indicators) | Missing (12.24%) |
| Father receiving income support (2 dummy indicators) | Missing (12.24%) |

9. The positive predicted values reported seems quite high and does not appear correct based on other information reported in the paper. Based on Table 3 the positive predicted value of the 95th percentile for the 90-day outcome should be 35% but in Table 2 it is reported as 51%

**Reply:** We have double checked the numbers and they are correct. The reviewer may have not read Table 2 correctly - the positive predictive value of the 95th percentile for the 90-day outcome is 34.9% there (Table 3 has been moved to the supplementary material as eTable 7).

**Table 2. Model performance metrics at various risk thresholds for predicting suicide attempt/death within 90 and 30 days following a visit to psychiatric specialty care during 2011–2012**

| Risk threshold | Sensitivity (%) | Specificity (%) | PPV (%) | NPV (%) |
| --- | --- | --- | --- | --- |
| Suicide attempt/death within 90 days following a visit | | | | |
| 99th | 17.5 | 99.6 | 64.6 | 96.9 |
| 98th | 27.7 | 99.0 | 51.2 | 97.3 |
| 97th | 36.5 | 98.3 | 45.0 | 97.6 |
| 96th | 42.0 | 97.5 | 38.9 | 97.8 |
| 95th | 47.2 | 96.6 | 34.9 | 97.9 |
| 90th | 62.1 | 92.0 | 23.0 | 98.4 |
| 85th | 71.5 | 87.2 | 17.6 | 98.8 |
| 80th | 77.4 | 82.2 | 14.3 | 99.0 |
| Suicide attempt/death within 30 days following a visit | | | | |
| 99th | 21.1 | 99.4 | 37.3 | 98.6 |
| 98th | 33.6 | 98.6 | 29.7 | 98.8 |
| 97th | 42.6 | 97.7 | 25.1 | 99.0 |
| 96th | 48.1 | 96.8 | 21.2 | 99.0 |
| 95th | 52.9 | 95.9 | 18.7 | 99.1 |
| 90th | 67.7 | 91.0 | 12.0 | 99.4 |
| 85th | 75.4 | 86.1 | 8.9 | 99.5 |
| 80th | 80.7 | 81.1 | 7.1 | 99.6 |

PPV: Positive predictive value; NPV: Negative predictive value

10. In all figures what model is being described should be indicated in the caption.

**Reply:** We have clarified in the caption that the models being described are the ensemble models.

11. It is unclear how the ensemble methods are put together. The weights associated with each model in the ensemble model combining all 4 top-performing models should be reported. Based on the results, it looks to me like the ensemble results are mostly driven by the gradient boosting model.

**Reply:** We assigned equal weights to the top-performing models. This has been described in the section of model derivation and evaluation **on page 8 line 13**:

*“We then compared the performance of the best models trained by the four algorithms, together with the ensemble models that used the average predicted risk of two or more of the best models for making prediction.”*

12. I would like to see the number of people and number of visits in each of the risk thresholds. I find it very surprising that the risk of suicide attempt, both predicted and observed is nearly 1 for anyone in the sample. Please indicate in the caption of the plot what the dots are in the calibration plot.

**Reply:** We assume that the reviewer is questioning why the dots in the calibration curves fall closely alongside the diagonal. We would like to clarify how the calibration curves were generated. In machine learning models, the raw probability generated directly by the model may poorly reflect the observed risk. Isotonic regression can be used to calibrate the raw probability so that the calibrated probability can better reflect the observed risk. The calibrated probability then will replace the raw probability and be used as predicted risk. In the current study, the calibration curves were generated after applying isotonic regression to the raw probability.

Specifically, the test set was first separated into a certain number of bins, the mean predicted risks (X-axis) and the proportion of observed positives (Y-axis) within each bin were then calculated and plotted.

The calibration curves were generated automatically by using the python code here (<https://scikit-learn.org/stable/auto_examples/calibration/plot_calibration_curve.html#sphx-glr-auto-examples-calibration-plot-calibration-curve-py>), with isotonic regression as the method for calibration. We are willing to share the code for the purpose of transparency.

Reviewer #4: "Predicting suicide attempt/death following a visit to psychiatric specialty care by applying machine learning to the Swedish national registry data" trained and validated various machine learning models, on a comprehensive Swedish dataset of 541,300 visits from 126,205 patients. The final ensemble model achieved AUCs of about 0.88-0.89, on predicting 30-day and 90-day suicide attempts. Various ancillary analyses were made to establish the degree of calibration, impact of additional training data and of the electronic medical records predictor subset.

In particular, the claimed PPVs from the validated model are very promising (0.349/0.187, with corresponding sensitivity of 0.472/0.529, at the 95th percentile risk threshold for 90-day/30-day outcomes respectively); a recent review (Belsher et al, JAMA Psychiatry, citation 6 in the manuscript) reported that most previous prediction models had extremely low PPVs of <0.01. That said, it should be noted that expected PPV appears to be significantly affected by the population the model(s) are applied to, with general populations with very low attempts/mortality being particularly challenging. As such, the performance achieved in this manuscript might be considered in the context of the target population already seeking psychiatric specialty care. Nonetheless, it remains an interesting study from the scale involved, and the comprehensiveness of the data features available, made possible by the quality of the Swedish national registers. Separation between training and test data also seems well-respected.

There however remain a number of issues that might be addressed:

1. While the study focuses on various machine learning models for data analysis (in particular: elastic net penalized logistic regression, random forest, gradient boosting [XGBoost], neural network), there are next to no pertinent details provided about these methods. For example, model hyperparameters are acknowledged as being grid-searched over, but there is no mention about what these hyperparameters were, nor their ranges (e.g. for random forests, how many trees, what were the tree max depths, etc? for neural networks, what was the architecture, activation functions, learning rate used, etc?) Although the datasets cannot be made public, there appears no reason why a reproducible description of the machine learning models cannot be described in the supplementary material.

**Reply:** We have provided information on the process of the optimization of hyperparameters using grid search in eTable 6. The code has been placed in a repository on Github (https://github.com/qichense/suicide_ml/).

2. The detailed training and validation AUC results presented in eTable 5 suggests that the relatively high PPV/AUC performance is largely due to the specific data examined, and less the specific state-of-the-art machine learning models used. In particular, logistic regression alone (albeit elastic net penalized, EN) achieved validation AUCs of 0.8721/0.8883 for 90-day/30-day suicide attempt prediction. The improvement with an ensemble of all four models (to 0.8751/0.8909 respectively) is extremely small. As such, it appears reasonable for the authors to focus on EN here, given it maintains the interpretability of standard logistic regression (e.g. via odds ratios; refer for example "Machine learning algorithms for outcome prediction in (chemo)radiotherapy: An empirical comparison of classifiers", Deist et al., Med Phys 2018), as opposed to the other methods.

**Reply:** We agree with this reviewer on two fronts – the improvement using ensemble methods is very small and the focus on elastic net penalized logistic regression allows for some interpretability. Meanwhile, we consider the similarity in model performance between the selected models an important finding of the current study, as this was not known until all planned analyses were done. The finding further adds to our conclusion that more informative predictors will be likely needed to further improve the predictive performance. Future research, using the same predictors as in our study, may employ elastic net penalized logistic regression only. Other the other hand, if more predictors are added, it might be premature to conclude that other model algorithms are unlikely to achieve better performance than the elastic net penalized logistic regression.

We have added the following text to the discussion **on page 13 line 15**:

*“…Future research using the same predictors as in our study may employ elastic net penalized logistic regression only to obtain a relatively better model interpretability at a limited loss of predictive performance.”*

3. It appears that the self-harm predictors were binary ("Age at the visit and family income were treated as continuous variables and rescaled to the range of 0-1. The other predictors were treated as binary or categorical variables"); was information about the frequency of self-harm episodes within each time period available, and if so, might this frequency be reasonably considered?

**Reply:** We agree with the reviewer that the frequency of self-harm episodes within each time period could be an important predictor. However, adding new variables would mean to retrain all models. We therefore want to stick to our project plan rather than add new predictors at this stage.

We have added the following text to the discussion **on page 13 line 19**:

*“Informative predictors could come from creating transformations of existing predictors (e.g., the frequency of self-harm over a certain time period) or incorporating completely novel predictors…”*

4. Given the greater importance of PPV due to the large number of negatives, the authors might consider exploring precision-recall curves and reporting the AUPRC metric too (e.g. alongside the ROC curves in Figure 1).

**Reply:** The ROC curves are presented in Figure 1 because the model selection was based on optimizing the metric of area under the ROC curve. Presenting the precision-recall curve alongside the ROC curves is therefore inappropriate in our view. We have considered presenting the prediction-recall curve alongside the Table 2 in which sensitivity, specificity, and predictive values were reported at varying risk thresholds. Our concern however is that the precision-recall curves may lead to that the readers neglect the importance of specificity and NPV by focusing only on PPV and sensitivity.

We therefore enclosed the precision-recall curves and AUPRC in the current response letter only and we are open for further discussion regarding whether to present the curves or not in the paper.

5. For Table 1, the figures in brackets might be explained.

**Reply:** Table 1 has been modified to make this clearer - figures in brackets represent percentages.

6. For eFigure 1, the sum of first-stage exclusion (1028907+210250) and qualifying visits after that exclusion (570945) does not appear to tally with the original total (1810602).

**Reply:** Thank you for spotting this mistake. It has now been corrected.

7. For eTable 1, the number of predictors accounted for at finer levels of granularity might also be listed, for convenience (e.g. "Diagnosis at the visit [37 predictors?]")

**Reply:** We have now added the number of predictors accounted for at finer levels of granularity in eTable 1.

8. eTable 6 further suggests that the bulk of predictive performance might be attained from a relatively small subset of predictors. In particular, "Intentional self-harm 3-6 months before the visit" was the top predictor for nearly all models, with "Family history of intentional self-harm" and "Intentional self-harm 1-3 months before the visit" probably the next most consistently important predictors. Given this, the authors might examine whether predictors relating to individual/family history of self-harm alone would yield comparable performance to the full 425-predictor models, or indeed the electric medical record-only predictor subsets (which again are able to achieve similar AUCs of 0.86/0.88).

**Reply:** This is a very good point and it deserves investigating in a separate study. Moreover, it would be not be necessary to use complicated machine learning algorithms to model so few predictors (i.e., individual/family history of self-harm). Ordinary logistic regression will likely work as well. Given that the current study did not seek to compare the performance of machine learning models and ordinary logistic regression, such analyses are beyond the scope of the study in our view. Nevertheless, we agree that the top-ranked predictors such as individual and family history of self-harm detected by more than one model point to the importance of investigating and quantifying the predictive power of these predictors in future research.

We have added to the discussion **on page 15 line 23**:

*“future research could compare ordinary logistic regression models with machine learning models, and in particular the former can focus on the top ranked predictors identified in this study.”*

9. The authors might consider providing another table with the data from eTable 6, but sorted by (mean?) rank to show predictors in general order of importance.

**Reply:** Thank you for this comment. We agree that the results should be presented in a more informative way. We are not sure sorting by mean rank is the correct way to present this, given considerable difference in rank of predictor importance between the models. We instead provided a new Table (Table 3) with the predictors sorted roughly by the rank of their importance, while taking into account both the rank of predictor importance by different models and the overlap of chosen predictors between these models.

**Table 3. Predictors ranked top 30 by the best models of elastic net penalized logistic regression, random forest, and gradient boosting**

| **Predictor** | **EN_90_** | **RF_90_** | **GB_90_** | **EN_30_** | **RF_30_** | **GB_30_** |
| --- | --- | --- | --- | --- | --- | --- |
| Intentional self-harm 3–6 months before the visit | 1^st^ | 2^nd^ | 1^st^ | 1^st^ | 1^st^ | 1^st^ |
| Intentional self-harm within 1 month before the visit | 2^nd^ | 7^th^ | 6^th^ | 2^nd^ | 5^th^ | 2^nd^ |
| Intentional self-harm 1–3 months before the visit | 3^rd^ | 5^th^ | 3^rd^ | 3^rd^ | 2^nd^ | 3^rd^ |
| Unplanned visit | 4^th^ | 17^th^ | 14^th^ | 8^th^ | 14^th^ | 12^th^ |
| Family history of intentional self-harm | 5^th^ | 1^st^ | 2^nd^ | 6^th^ | 3^rd^ | 5^th^ |
| Intentional self-harm 6–12 months before the visit | 6^th^ | 3^rd^ | 5^th^ | 7^th^ | 4^th^ | 7^th^ |
| Prior intentional self-harm by sharp object | 7^th^ | 8^th^ | 7^th^ | 4^th^ | 9^th^ | 6^th^ |
| Prior intentional self-harm by poisoning | 8^th^ | 4^th^ | 4^th^ | 5^th^ | 6^th^ | 4^th^ |
| Unplanned visit 1–3 months before the visit | 9^th^ | 9^th^ | 10^th^ | 12^th^ | 12^th^ | 11^th^ |
| Unplanned visit within 1 month before the visit | 11^th^ | 6^th^ | 12^th^ | 9^th^ | 7^th^ | 9^th^ |
| Hospitalization within 1 month before the visit | 12^th^ | 12^th^ | 11^th^ | 11^th^ | 8^th^ | 10^th^ |
| Family history of substance use disorder | 13^th^ | 23^rd^ | 17^th^ | 17^th^ | 23^rd^ | 25^th^ |
| Family history of borderline personality disorder | 14^th^ | 16^th^ | 13^th^ | 14^th^ | 15^th^ | 8^th^ |
| Intentional self-harm 1–3 years before the visit | 15^th^ | 10^th^ | 8^th^ | 16^th^ | 10^th^ | 16^th^ |
| Family history of self-harm of undetermined intent | 16^th^ | 24^th^ | 21^st^ | 15^th^ | 24^th^ | 23^rd^ |
| Hospitalization 1–3 months before the visit | 19^th^ | 11^th^ | 9^th^ | 19^th^ | 13^th^ | 14^th^ |
| In/out-patient visit | 27^th^ | 15^th^ | 16^th^ | 27^th^ | 16^th^ | 20^th^ |
| Family history of anxiety disorders | 26^th^ | 30^th^ | 20^th^ | 22^nd^ | … | 30^th^ |
| Planned visit | … | 18^th^ | 18^th^ | 10^th^ | 17^th^ | 13^th^ |
| Age at the visit | 10^th^ | … | 26^th^ | 13^th^ | … | … |
| Diagnosis of major depressive disorder within 1 month before the visit | 17^th^ | … | 24^th^ | 24^th^ | … | … |
| Sex | 21^st^ | … | 30^th^ | 23^rd^ | … | … |
| Family history of major depressive disorder | 24^th^ | … | 27^th^ | 25^th^ | … | … |
| Diagnosis of anxiety within 1 month before the visit | 18^th^ | … | … | 20^th^ | … | … |
| Intentional self-harm at the visit | 20^th^ | … | … | 18^th^ | … | … |
| Diagnosis of ADHD at the visit | 22^nd^ | … | … | 26^th^ | … | … |
| Presence of study income | 23^rd^ | … | … | 29^th^ | … | … |
| Dispensed benzodiazepines 6–12 months before the visit | 25^th^ | … | … | … | … | … |
| Diagnosis of other personality disorders than ASPD and BLPD 6–12 months before the visit | 28^th^ | … | … | … | … | … |
| Diagnosis of substance use disorder within 1 month before the visit | 29^th^ | … | … | … | … | … |
| Dispensed benzodiazepines within 1 month before the visit | 30^th^ | … | … | … | … | … |
| Diagnosis of borderline personality disorder within 1 month before the visit | … | 13^th^ | 19^th^ | … | 11^th^ | 15^th^ |
| Diagnosis of borderline personality disorder 6–12 months before the visit | … | 14^th^ | 23^rd^ | … | 18^th^ | … |
| Diagnosis of borderline personality disorder 1–3 months before the visit | … | 20^th^ | 15^th^ | … | 22^nd^ | 26^th^ |
| Diagnosis of borderline personality disorder 3–6 months before the visit | … | 21^st^ | 22^nd^ | … | 19^th^ | 19^th^ |
| Diagnosis of borderline personality disorder at the visit | … | 22^nd^ | 29^th^ | … | 21^st^ | … |
| Diagnosis of borderline personality disorder 1–3 years before the visit | … | 25^th^ | 25^th^ | … | 27^th^ | … |
| Intentional self-harm 3–5 years before the visit | … | 19^th^ | … | … | 20^th^ | … |
| Prior self-harm by poisoning of undetermined intent | … | 26^th^ | … | … | 26^th^ | 27^th^ |
| Diagnosis of other borderline personality disorders than ASPD and BLPD at the visit | … | 27^th^ | … | … | … | … |
| Diagnosis of substance use disorder 3–6 months before the visit | … | 28^th^ | … | … | 30^th^ | … |
| Diagnosis of borderline personality disorder 3–5 years before the visit | … | 29^th^ | … | … | 29^th^ | … |
| Dispensed antidepressants 3–6 months before the visit | … | … | 28^th^ | … | … | … |
| Dispensed anxiolytics 6–12 months before the visit | … | … | … | 21^st^ | … | … |
| Dispensed antipsychotics 1–3 years before the visit | … | … | … | 28^th^ | … | … |
| Diagnosis of epilepsy 3–6 months before the visit | … | … | … | 29^th^ | … | … |
| Father’s education £ 9 years | … | … | … | 30^th^ | … | … |
| Family history of other borderline personality disorders than ASPD and BLPD | … | … | … | … | 25^th^ | … |
| Intentional self-harm by unspecified means | … | … | … | … | 28^th^ | … |
| Diagnosis of substance use disorder 6–12 months before the visit | … | … | … | … | … | 17^th^ |
| Diagnosis of other personality disorders than ASPD and BLPD 1–3 months before the visit | … | … | … | … | … | 18^th^ |
| Diagnosis of type 2 diabetes mellitus 1–3 months before the visit | … | … | … | … | … | 21^st^ |
| Diagnosis of asthma 3–5 years before the visit | … | … | … | … | … | 22^nd^ |
| Diagnosis of substance use disorder at the visit | … | … | … | … | … | 24^th^ |
| Diagnosis of epilepsy within 1 month before the visit | … | … | … | … | … | 28^th^ |

EN: The best elastic net penalized logistic regression model

RF: The best random forest model

GB: The best gradient boosting model

ASPD: Antisocial personality disorder

BLPD: Borderline personality disorder

10. In the Model derivation and evaluation section, it is stated that "Based on the results of cross-validation, the model giving the highest average validation AUC and showing the smallest difference between the training and validation AUCs was selected and applied to the entire training set to obtain the final model parameters". However, the model having the highest average validation AUC might not necessarily also show the small difference between training and validation AUCs. How were these objectives balanced?

**Reply**: We thank the reviewer for pointing this out. We have now rephrased the sentence as follows **on page 8 line 15**:

*“Based on the results of cross-validation, among the models giving the highest average validation AUC (****values unchanged to the fourth decimal place after rounding were considered the same****), the one showing the smallest difference between the training and validation AUCs was selected and applied to the entire training set to obtain the final model parameters”*

11. A "risk threshold" at various percentiles is referenced frequently, and appears to be achievable by varying the operating point of each machine learning model. There might be a brief explanation of what the various percentiles of risk threshold signify.

**Reply:** This is a helpful comment, and we agree that some explanation would be helpful to readers. The 90^th^, 95^th^, 99^th^ percentile risk thresholds are conventionally selected thresholds for comparison between previous studies (4). We chose to report the model performance metrics at varying risk thresholds to show how these metrics vary across threshold. In the revised manuscript, we have reported the metrics additionally at 99.5^th^ , 70^th^, 60^th^, 50^th^ to provide a more comprehensive picture of the metrics varying across risk thresholds (Table 2).

12. Certain assertions appear too definite, e.g. "Because the validation curve is no longer increasing with increased training sample size, we can conclude that future improvements to the model will require more informative predictors and higher model capacity rather than a larger sample size".

**Reply:** On re-reading, we agree and have rephrased the sentence **on page 10 line 21**:

*"Because the validation curve is no longer increasing with increased training sample size, future improvements to the model may require more informative predictors and higher model capacity rather than a larger sample size".*

13. Some analysis/comment on prediction of attempts vs. prediction of deaths would be interesting.

**Reply:** We have added the following text to the discussion **on page 15 line 19**:

*“In our study, the models were optimized to predict suicidal behaviors rather than the rare outcome of suicide deaths. In relation to deaths, the model performance would be quite different with higher specificities and lower sensitivities, and the calibration would be poor. As some metrics of discrimination will be low, one promising approach would be to solely use probability scores rather than risk thresholds to investigate suicide deaths.”*

14. Finally, there are some minor textual issues:

"The Medical Birth Register covers nearly all deliverys in Sweden..." -> "nearly all deliveries"

"Despite statistically significant decrease in the value (p<0.05)." -> incomplete sentence fragment?

**Reply:** The typo has been corrected. The incomplete sentence has been rephrased as follows **on page 11 line 15**:

*“Compared with the main models, these decreases in AUC were statistically significant (p<0.05).”*

In summary, while the authors have systematically investigated various ensemble combinations of machine learning models on a large set of 425 predictors in their main analyses, the presented evidence suggests that virtually the same performance might have been achieved with just logistic regression and a much smaller set of the most informative predictors relating to individual/family history of self-harm. Therefore, while the contribution to suicide prediction remains welcome, the applicability of less-interpretable machine learning models (random forests, XGBoost, neural networks) seems not yet well established, for this particular study.

**Reply:** We agree with the reviewer that based on the results, the elastic net penalized logistic regression seem to have a predictive performance not obviously worse than the other models. Meanwhile, we consider the similarity in model performance between the selected models an important finding of the current study, as this could not be known until all planned analyses were done. The finding further adds to our conclusion that more informative predictors are likely needed to further improve the predictive performance. Future research, using the same predictors as in our study, may employ elastic net penalized logistic regression only. Other the other hand, if more predictors are added, it might be premature to conclude that other model algorithms are unlikely to achieve better performance than the elastic net penalized logistic regression.

We have added the following text to the discussion **on page 13 line 15**:

*“…Future research using the same predictors as in our study may employ elastic net penalized logistic regression only to obtain a relatively better model interpretability at a limited loss of predictive performance.”*

**Reviewer #5:** The authors applied an ensemble learning method including four types of models to predict suicide attempt/death within a 90-days/30-days window for patients aged 18-39 after a visit to psychiatric specialty care using Swedish national registry data and electronic health records (EHR). 425 candidate predictors were carefully selected covering all relevant information in the registry. The models were trained using a large sample size of patient visits and model parameters were selected using cross-validation. Results showed that the ensemble model outperformed existing models on discrimination and calibration, with high AUC, specificity and negative predictive values. Main findings include a comparison of top 30 predictors from each model, and prediction result using only EHR data also had good performance.

Strength: The study investigated an urgent and important topic in mental health. The authors applied a suite of machine learning methods to high quality national registry data. Analyses were well done with clear communication of their findings. The top predictors could provide insightful information to practitioners.

Weakness: The developed ensemble model has not been externally validated. So it is unclear how well it will perform prospectively or in a different patient population. Sensitivity and positive predictive values are relatively low, which decrease the potential impact of this model as a prevention/monitoring tool.

I have the following comments to the authors:

1. It is unclear why these four particular types of machine learning models were selected in this study. Could you provide a brief rationale and explanation of the advantage/disadvantage of each type of model? Since they performed similarly, why include all four models?

**Reply:** Thank you for these thoughtful comments. We agree, in retrospect, that a brief rationale is currently missing from the paper. We would like to clarify that our study aimed to explore the potential of the Swedish registry data in predicting suicidal behavior using machine learning approaches. Due to limited knowledge on the topic, the performance of the machine learning models on the data was largely unpredictable before the analyses were done. The selection of model algorithms to be tested was somewhat arbitrary. Ideally, we would like to test all existing machine learning algorithms on the same data, but that would be extremely time consuming. For practical purposes, we therefore selected four relatively popular model algorithms to start with. The ensemble method then could make use of the diversity in analytic approach between these algorithms.

We have added a brief rationale for model selection **on page 8 line 7**:

*“The four model algorithms were selected, first, because they have been repeatedly used in previous research but have never been applied to the same data in the same study; second, because the models diverse in analytic approach, which makes it possible to be aggregated using an ensemble method to achieve a predictive performance better than each individual model.”*

2. It is unclear how missing data on the predictors were handled in the study. Could you provide the procedure for handling missing data? Were there any imputation done?

**Reply:** When describing predictors in the Method section, we have clarified that “Missingness on the predictors ranged from 0.6% to 12.5% (eTable 1 in the online supplement) and was coded as a separate category for each predictor.” No imputation was done, because missingness on several variables tended to occur to the same individuals and therefore the assumption of missing at random (MAR) for imputation method was apparently violated. We have therefore added the following text **on page 7 line 24**:

*“Missing predictors appeared to co-occur in the same person. Therefore, no imputation was done as missingness at random could not be assumed.”*

3. Why did you select 80th-99th risk thresholds in Table 2?

**Reply:** The selection was somewhat arbitrary. We thought that any risk calculator would not be feasible if it led to large numbers of people identified at high risk, and we thought that 80th percentile and above was a reasonable threshold that balanced the clinical feasibility with model performance. In the revised manuscript, we have reported the metrics at 99.5^th^–50^th^ percentile risk thresholds to provide a better picture of the prediction performance.

**Table 2. Model performance metrics at various risk thresholds for predicting suicide attempt/death within 90 and 30 days following a visit to psychiatric specialty care during 2011–2012**

| **Risk threshold** | **Sensitivity (%)** | **Specificity (%)** | **PPV (%)** | **NPV (%)** |
| --- | --- | --- | --- | --- |
| *Suicide attempt/death within 90 days following a visit* | | | | |
| 99.5^th^ | 10.0 | 99.9 | 74.2 | 96.7 |
| 99^th^ | 17.5 | 99.6 | 64.6 | 96.9 |
| 98^th^ | 27.7 | 99.0 | 51.2 | 97.3 |
| 97^th^ | 36.5 | 98.3 | 45.0 | 97.6 |
| 96^th^ | 42.0 | 97.5 | 38.9 | 97.8 |
| 95^th^ | 47.2 | 96.6 | 34.9 | 97.9 |
| 90^th^ | 62.1 | 92.0 | 23.0 | 98.4 |
| 85^th^ | 71.5 | 87.2 | 17.6 | 98.8 |
| 80^th^ | 77.4 | 82.2 | 14.3 | 99.0 |
| 70^th^ | 85.5 | 72.1 | 10.6 | 99.2 |
| 60^th^ | 90.5 | 61.9 | 8.4 | 99.4 |
| 50^th^ | 94.0 | 51.7 | 7.0 | 99.6 |
| *Suicide attempt/death within 30 days following a visit* | | | | |
| 99.5^th^ | 12.8 | 99.7 | 44.9 | 98.5 |
| 99^th^ | 21.1 | 99.4 | 37.3 | 98.6 |
| 98^th^ | 33.6 | 98.6 | 29.7 | 98.8 |
| 97^th^ | 42.6 | 97.7 | 25.1 | 99.0 |
| 96^th^ | 48.1 | 96.8 | 21.2 | 99.0 |
| 95^th^ | 52.9 | 95.9 | 18.7 | 99.1 |
| 90^th^ | 67.7 | 91.0 | 12.0 | 99.4 |
| 85^th^ | 75.4 | 86.1 | 8.9 | 99.5 |
| 80^th^ | 80.7 | 81.1 | 7.1 | 99.6 |
| 70^th^ | 87.9 | 71.0 | 5.2 | 99.7 |
| 60^th^ | 92.6 | 60.9 | 4.1 | 99.8 |
| 50^th^ | 95.2 | 50.8 | 3.4 | 99.8 |

PPV: Positive predictive value; NPV: Negative predictive value

Model performance metrics were based on ensemble models

4. The predictors listed in eTable 6 are very important results and should not be in the appendix. How about swapping with Table 3 or Figure 3 which is not very interesting?

**Reply:** We agree that the information in eTable 6 is of importance. We have now presented the information as Table 3 in the main text.

5. The paper is written for an audience with background in machine learning and know the concepts of model discrimination and calibration. It would be helpful to include the definition of these concepts when they first appear for a broader readership. For example, the explanations on sensitivity and specificity in 3rd paragraph on page 10 should appear earlier in the paper.

**Reply:** This is a helpful comment, and we agree. We have clarified the concepts of discrimination and calibration in the Methods; we have moved the explanations on sensitivity and specificity to the Methods as well. We have also added the following to clarify the AUC meaning **on page 8 line 21**:

*“The AUC for the test set was used to evaluate model discrimination (****i.e., the extent to which a prediction model can separate those who will experience suicidal events from those who will not****). … …The Brier score (equal to zero under perfect calibration), along with calibration plots, was used to assess model calibration (i.e., the agreement between observed proportion of positives and mean predicted risk of the outcome in different risk strata). ”*

6. The last sentence on page 9, starting with "Despite statistically…", is unclear what it is referring to.

**Reply:** Agreed. The sentence has been rephrased as follows **on page 11 line 15**:

*“Compared with the main models, these decreases in AUC were statistically significant (p<0.05).”*

**References**

1.	Sariaslan A, Sharp DJ, D'Onofrio BM, Larsson H, Fazel S. Long-Term Outcomes Associated with Traumatic Brain Injury in Childhood and Adolescence: A Nationwide Swedish Cohort Study of a Wide Range of Medical and Social Outcomes. PLoS Med. 2016;13(8):e1002103.

2.	Ludvigsson JF, Lu D, Hammarstrom L, Cnattingius S, Fang F. Small for gestational age and risk of childhood mortality: A Swedish population study. PLoS Med. 2018;15(12):e1002717.

3.	Ludvigsson JF, Andersson E, Ekbom A, Feychting M, Kim JL, Reuterwall C, et al. External review and validation of the Swedish national inpatient register. BMC Public Health. 2011;11:450.

4.	Belsher BE, Smolenski DJ, Pruitt LD, Bush NE, Beech EH, Workman DE, et al. Prediction Models for Suicide Attempts and Deaths: A Systematic Review and Simulation. JAMA psychiatry. 2019.
